# Functional Analysis of the *teosinte branched 1* Gene in the Tetraploid Switchgrass (*Panicum virgatum L*.) by CRISPR/Cas9-Directed Mutagenesis

**DOI:** 10.3389/fpls.2020.572193

**Published:** 2020-09-23

**Authors:** Yang Liu, Weiling Wang, Bing Yang, Christopher Currey, Shui-zhang Fei

**Affiliations:** ^1^Interdepartmental Program in Plant Biology, Iowa State University, Ames, IA, United States; ^2^Department of Horticulture, Iowa State University, Ames, IA, United States; ^3^Innovation Center of Rice Cultivation Technology in Yangtze River Valley, Ministry of Agriculture/Key Laboratory of Crop Genetics and Physiology of Jiangsu Province, Yangzhou University, Yangzhou, China; ^4^Christopher S. Bond Life Sciences Center, Division of Plant Sciences, University of Missouri, Columbia, MO, United States; ^5^Donald Danforth Plant Science Center, St. Louis, MO, United States

**Keywords:** CRISPR/Cas9, gene editing, *Teosinte branched 1*, tillering, micropropagation, switchgrass

## Abstract

Tillering is an important biomass yield component trait in switchgrass (*Panicum virgatum L*.). *Teosinte branched 1* (*tb1*)/*Branched 1* (*BRC1*) gene is a known regulator for tillering/branching in several plant species; however, its role on tillering in switchgrass remains unknown. Here, we report physiological and molecular characterization of mutants created by CRISPR/Cas9. We successfully obtained nonchimeric *Pvtb1a* and *Pvtb1b* mutants from chimeric T0 mutants using nodal culture. The biallelic *Pvtb1a-Pvtb1b* mutant plants produced significantly more tillers and higher fresh weight biomass than the wild-type plants. The increased tiller number in the mutant plants resulted primarily from hastened outgrowth of lower axillary buds. Increased tillers were also observed in transgene-free BC1 monoallelic mutants for either *Pvtb1a-Pvtb1b* or *Pvtb1b* gene alone, suggesting *Pvtb1* genes act in a dosage-dependent manner. Transcriptome analysis showed 831 genes were differentially expressed in the *Pvtb1*a-*Pvtb1b* double knockdown mutant. Gene Ontology analysis revealed downregulation of *Pvtb1* genes affected multiple biological processes, including transcription, flower development, cell differentiation, and stress/defense responses in edited plants. This study demonstrates that *Pvtb1* genes play a pivotal role in tiller production as a negative regulator in switchgrass and provides opportunities for further research aiming to elucidate the molecular pathway regulating tillering in switchgrass.

## Introduction

Switchgrass (*Panicum virgatum*), a C4 perennial grass with demonstrated high biomass yield is native to North America and is well adapted to marginal land not suitable for food crops (Mitchell et al., 2008; Narasimhamoorthy et al., 2008). The low production cost and high lignocellulose-based biofuel potential make it well-suited for bioenergy crop (McLaughlin and Kszos, 2005). It was named the model species for herbaceous bioenergy crop by the U.S. Department of Energy in 1991 following more than a decade of research (Wright and Turhollow, 2010). Switchgrass is an out-crossing species with varied ploidy levels and is self-incompatible with individual plants being highly heterozygous (Narasimhamoorthy et al., 2008). Upland and lowland ecotypes are the two major representative taxa of switchgrass (Zhang et al., 2011). Most of the low-tillering lowland ecotypes are tetraploid (2n=4x=36), while the high-tillering upland ecotypes contain both tetraploids and octoploids (2n=8x=72) with hexaploids (2n=6x=54) being reported rarely (Zhang et al., 2011). Unlike the lowland ecotype, which has a caespitose growth habit and poor freezing tolerance, upland ecotypes are highly rhizomatous and cold hardy (Mitchell and Schmer, 2012).

High biomass yield is a high priority for switchgrass breeding (Casler, 2010; Okada et al., 2010; Lu et al., 2013). Tiller number is positively correlated with biomass yield (Das et al., 2003; Boe, 2007; Boe and Beck, 2008). Therefore, understanding the molecular mechanism regulating tillering in switchgrass can facilitate development of high-yielding cultivars. Tillers result from the outgrowth of axillary buds at nodes in grass species and the number of tillers produced from a single plant varies greatly by species and genotypes. Tillering or branching is regulated by numerous endogenous and environmental factors in both eudicots and monocots (Kebrom et al., 2010; Whipple et al., 2011; Reddy and Finlayson, 2014; Gonzalez-Grandio et al., 2017; Holalu and Finlayson, 2017). For instance, auxin is a well-known contributor to apical dominance, which inhibits axillary bud outgrowth, while cytokinins (CKs) promote bud outgrowth (Thimann and Skoog, 1933; Morris, 1977; Minakuchi et al., 2010; Braun et al., 2012). Strigolactones (SLs), another key plant hormone, represses branching or tillering in both monocots and eudicots (Sorefan et al., 2003; Zou et al., 2006; Arite et al., 2007). Additionally, tiller production is often inhibited under severe shade (Kebrom et al., 2006; Finlayson et al., 2010; Kebrom et al., 2010), insufficient mineral nutrients or photosynthates (Wang et al., 2019).

The *teosinte branched 1* (*tb1*) gene is an important tillering/branching-related transcription factor gene that regulates tillering by integrating environmental and developmental cues (Doebley et al., 1997; Whipple et al., 2011; Seale et al., 2017). In cultivated maize (*Zea mays* L.), an insertion in the regulatory region of the *tb1* gene elevates its expression at leaf axils where axillary buds are located and greatly suppresses tillering, while its ancestor teosinte lacking the insertional sequence is highly branched (Studer et al., 2011). *tb1* belongs to the *TCP* (*TEOSINTE BRANCHED1*, *CYCLOIDEA*, *PCF*) gene family that all encode proteins with a 59-amino acid, noncanonical basic helix-loop-helix (bHLH) motif that allows DNA binding and protein-protein interactions (Martin-Trillo and Cubas, 2010). Closely related *tb1* genes with similar functions have been identified in both monocots and dicots (Kebrom et al., 2006; Aguilar-Martinez et al., 2007; Martin-Trillo et al., 2011; Choi et al., 2012; Nicolas et al., 2015). For example, the *OsTB1* gene functions as a negative regulator for lateral branching in rice (*Oryza sativa* L.) (Takeda et al., 2003; Choi et al., 2012). The ortholog of *tb1* in bread wheat (*Triticum aestivum* L.) regulates inflorescence architecture and outgrowth of axillary buds (Dixon et al., 2018). In Arabidopsis, two orthologs of *tb1*, *BRANCHED 1* (*BRC1*) and *BRANCHED 2* (*BRC2*), regulate branching with *BRC1* having the major effect (Aguilar-Martinez et al., 2007). Many studies demonstrate *tb1* genes integrate multiple signaling pathways to regulate bud outgrowth and tillering (Kebrom et al., 2013; Rameau et al., 2015). Low ratio of red light to far-red light (R/FR), as a result of shade, inhibits the outgrowth of axillary buds in sorghum (*Sorghum bicolor* (L.) Moench) by upregulating the expression of *SbTB1*, an ortholog of *tb1* (Kebrom et al., 2006; Kebrom et al., 2010). In Arabidopsis (*Arabidopsis thaliana* (L.) heynh.), *BRC1* suppresses lateral bud growth in response to shade by promoting abscisic acid accumulation inside axillary buds (Gonzalez-Grandio et al., 2017). Cytokinins activate axillary bud outgrowth by inhibiting localized *PsBRC1* expression in pea (*Pisum sativum* L.) (Braun et al., 2012) or *OsTB1* in rice (Minakuchi et al., 2010). These studies all demonstrate that *BRC1/TB1* is a common target for hormonal and environmental signals that regulate tillering/branching across species. Hence, understanding the function of switchgrass *tb1* genes can provide valuable information to the understanding of the tillering mechanisms in switchgrass.

The lowland switchgrass cv. Alamo from which the reference genome was produced is an allotetraploid (2n = 4x = 36, NNKK). It is therefore hypothesized that the majority of genes are present as homoeologs. There are two *Pvtb1* genes (*Pvtb1a* and *Pvtb1b*) in ‘Alamo’ with 89% DNA sequence identity between them (*Panicum virgatum* v4.1, DOE-JGI, http://phytozome.jgi.doe.gov/). Because of its self-incompatibility, it is difficult to obtain homozygous mutants in switchgrass by inbreeding hemizygous mutants as is done for self-pollinating transgenic plants. The clustered regularly interspaced short palindromic repeat (CRISPR)/CRISPR-associated protein 9 nuclease (Cas9) based genome editing tools have become a powerful genetic tool for gene function analysis or crop improvement (Zhao et al., 2016; Wang et al., 2018; Yang et al., 2018). A multiplex CRISPR/Cas9 platform can be used to simultaneously edit multiple genes, which is particularly advantageous for a polyploid species such as switchgrass in which homeologs of a same gene exist, or for members of a gene family that are present in tandem (Zhao et al., 2016).

We previously created switchgrass *tb1* mutant plants by using CRISPR/Cas9 (Liu et al., 2018). However, the allelic composition of *Pvtb1* genes in these T0 mutant plants was not fully characterized. In addition, it remained possible that these primary mutants are chimeric, which prevented us from accurately assessing the function of *tb1* genes. In plant species that can be vegetatively propagated, nonchimeric, solid mutants can be successfully isolated from chimeric mutants (Maluszynski et al., 1995). For example, separating mutated sectors from chimeric mutants by *in vitro* multiplication has been achieved in banana, cassava and other vegetatively propagated crops (Harten et al., 1981; Novák et al., 1990; Maluszynski et al., 1995; Das et al., 2000). Switchgrass can be readily propagated using node culture (Alexandrova et al., 1996). Hence, generation of primary mutants with CRISPR/Cas9, followed by nodal culture would allow us to generate nonchimeric mutants without the need of producing progeny.

The relative ease with which transgene-free mutant plants can be obtained is a unique advantage for CRISPR/Cas9-based genome editing. Transgene-free mutants have been obtained in multiple crops using CRISPR/Cas9-based genome editing tools and stable inheritance of CRISPR/Cas9 induced mutations has been demonstrated in other plant species, (Pyott et al., 2016; Zhang et al., 2016; He et al., 2018) but not yet in switchgrass. Germplasm generated through CRISPR/Cas9-based genome editing method without foreign DNAs may bypass or simplify the regulatory process required for crops created with traditional transgenic approaches (Waltz, 2018).

Here, we report the successful use of micropropagation to generate nonchimeric mutants from chimeric primary mutants induced by CRISPR/Cas9, eliminating the need of obtaining progeny mutants for gene function analysis. Moreover, the transmission of CRISPR/Cas9-induced mutations in switchgrass is demonstrated in this study. Transgene-free progeny mutants, preserving the phenotypic effect of *Pvtb1* mutations similar to that exhibited in T0 mutants, were generated in this study. We showed that double biallelic mutant for *Pvtb1a* and *1b* genes enhanced tiller production and increased biomass yield, indicating that *Pvtb1* genes negatively regulate tillering in switchgrass. Transcriptome analysis of a *Pvtb1* knockdown mutant 52-1 and wild-type (WT) plant WT-1 suggest that *Pvtb1* genes are involved in multiple pathways to regulate tillering in switchgrass.

## Materials and Methods

### Micropropagation and Seed Propagation of CRISPR/Cas9-Induced *Pvtb1* Mutants

Primary mutant plants were generated previously (Liu et al., 2018). Briefly, the CRISPR/Cas9 construct containing two gRNAs targeting the conserved regions of *Pvtb1a* and *Pvtb1b* genes was delivered into the genome of switchgrass through *Agrobacterium*-mediated transformation of caryopsis-derived embryogenic callus from which primary mutant plants were obtained. To produce nonchimeric mutants from chimeric primary mutants, we micropropagated *Pvtb1* mutants by culturing nodes containing axillary buds according to (Harten et al., 1981; Alexandrova et al., 1996). Each of the two basal nodes from a stem containing an axillary bud with a portion of (~1.5-cm long) internode above and below the node was selected for *in vitro* culture on the MS-0 medium with 3 gL^-1^ Phytagel (Sigma Chemical Co., St. Louis) and no plant growth regulator. These nodal segments were surface sterilized in commercial bleach (5% aqueous solution of sodium hypochlorite) for 30 min, and then rinsed three times with sterile distilled deionized water. Three experiments were conducted for the 52-1 and 35-2 mutants and their corresponding WT (WT-1) plants with 20 nodes for each experiment. After 8 weeks of culture in a growth chamber with a light intensity of 140 µmol m^-2^ s^-1^ at a photoperiod of 16/8 h light/dark and a temperature of 25°C, regeneration efficiency (regenerated plantlets/total nodes number) were calculated. After roots emerged, plantlets were grown in 6-inch diameter (1 L) pots using commercial soil mix (Sunshine soil mix #1, Sun Gro) of peat moss and perlite and moved into a mist room for 7–10 days before they were placed in a greenhouse at 26°C with a 16/8 h (day/night) photoperiod and a light intensity of approximately 400 µmol m^-2^ s^-1^.

To determine the inheritance of CRISPR/Cas9 induced mutations in switchgrass, BC1 progeny were obtained by crossing mutants with genetically compatible WT plants. Seeds were harvested from the mutant parents and were sowed in a 1,020 tray (54.5 cm L × 27.8 cm W × 6.2 cm H) with the same soil as described previously that was maintained at a mist room until germination. Seedlings were transferred to the same greenhouse as described earlier. The presence of the transgene was analyzed using PCR with gRNA/Cas9-specific primers (**Table S6**).

### Genotyping of CRISPR/Cas9-Induced *Pvtb1* Mutants by Next Generation Sequencing

NGS was used to genotype the *Pvtb1* mutant plants generated from micropropagation of primary mutants and BC1 *Pvtb1* mutant progeny. To amplify the target fragments of each *Pvtb1* gene, gene-specific primers were designed for *Pvtb1a* and *Pvtb1b* respectively. The amplicon size for *Pvtb1a* is 250 bp, while that of *Pvtb1b* is 288 bp, both are sufficiently long to cover the two target sequences. The Illumina overhang adapter sequences were added to the gene-specific primers (**Table S6**). A second round of PCR was used to add the dual indices with the Nextra XT Index Kit (Illumina, San Diego, CA, USA). The quality of these sequencing libraries was determined by the Qubit^®^ 2.0 Fluorometer. The 150-cycle HiSeq sequencing was done for all the amplicon libraries at the DNA Facility at Iowa State University (ISU). For each library, at least 5,000 reads were generated to determine the sequence of the respective amplicons. The results were analyzed by CRISPR-DAV pipeline (Wang et al., 2017).

### Sequence Alignment and Phylogenetic Analysis

The sequence of maize *tb1* gene was used to blast the sequences of *Pvtb1* genes from the *P. virgatum* genome project v4.1 (Panicum virgatum v1.0, DOE-JGI, http://phytozome.jgi.doe.gov/). The *Pvtb1* genes were subsequently isolated and sequenced with the *Pvtb1* gene-specific primers (**Table S6**). Full-length putative amino acid sequences of *tb1* gene orthologs were obtained from Phytozome (https://phytozome.jgi.doe.gov/pz/portal.html) (**Table S7**). Alignments of the putative protein sequences of TB1 were performed using Clustal Omega (Sievers et al., 2011) (https://www.ebi.ac.uk/Tools/msa/clustalo/).

### Phenotypic Characterization of *Pvtb1* Mutant Plants With Hydroponic Culture

To investigate the effects of *Pvtb1* genes on phenotype of switchgrass, we established a hydroponic system to observe plant growth. Nodal segments were cut from the wild-type plant (WT, genotype AABB) and mutant 52-1-3 (genotype aabb), 52-1-1 (genotype Aabb) plants, and cultured *in vitro* as described previously. Plants of similar height that were grown in 6-inch pots for 10 days were transferred into a hydroponic device in a greenhouse where temperature was maintained at 26°C with a 16h/8h (day/night) photoperiod with a light intensity of approximately 400 μmol m^-2^ s^-1^. For hydroponic culture, the stem base of each plant was wrapped around by sponge and placed into a small basket which was then inserted into a hole on the lid of a plastic container (51 cm L × 43 cm W × 15 cm H) that was filled with a nutrient solution (16 - 4 - 17 Oasis Hydroponic, JR Peters Inc., PA, USA). Electrical conductivity (EC) and pH of the nutrient solution were measured by a portable pH/EC/Temperature meter (HI9813-6, Hanna Instruments, Inc., RI, USA) daily and maintained at 1.35 mS cm^-1^ and 6.0, respectively. The pH was lowered or raised by phosphoric acid or sodium hydroxide, respectively, while the EC was maintained by adding concentrated fertilizer stock solution or clear water. The nutrient solution was aerated by an aquarium air pump (ActiveAQUA, Hydrofarm, CA, USA) to ensure adequate oxygen supply.

### Statistical Analysis of Morphological Traits

Primary, secondary, tertiary or quaternary tillers were visually determined according to Davidson and Chevalier (1987). Primary tillers were defined as those which arise from leaf axils in nodes of the main stem whereas secondary tillers arise from leaf axils in nodes of the primary tillers. By the same rule, tertiary and quaternary tillers were defined as those arise from leaf axils of nodes of secondary and tertiary tillers, respectively. Adventitious roots of newly formed tillers tend to have creamy white color whereas roots of older tillers appear in darker color. This color variation was used to assist classification of tillers. Plant height was determined by measuring the tallest tiller for each plant. Root length was determined by measuring the length of the longest root for each plant. Stem diameter was determined by the average diameter of the middle internode of five largest tillers for each plant. The number of tillers and roots (longer than 2 cm) were also counted. To determine biomass yield, pot-grown plants were manually cut at the ground level using a pruning shears and weighted for fresh weight. Samples were then oven-dried at 70°C for 4 days when they reached a constant weight to obtain the dry weight. Three to six biological replicates were used in each measurement. Student’s t-test was used to determine the significance of difference between the mutant plants and the WT plants.

### Transcriptome Sequencing and Analysis of the mRNA-Seq Data

It was shown in the expression database that *Pvtb1* genes in switchgrass expressed highly in axillary buds (Panicum virgatum v1.0, DOE-JGI, http://phytozome.jgi.doe.gov/). Thus, we extracted RNAs from axillary buds of tillers. The mutant 52-1 with highly enhanced tiller production and the WT plant (WT-1) generated from the same callus line from which the mutant 52-1 was obtained were chosen for mRNA-seq. Three biological replications were sampled for each plant. All visible axillary buds collected from two to three tillers are treated as one biological replicate. Total RNA was isolated from tissue samples using the Qiagen RNeasy Plant Isolation kit according to the manufacturer’s protocol (Qiagen Inc., Valencia, CA, USA) and the RNA quality was checked using BioAnalyzer 2100 (Agilent Technologies, Santa Clara, CA, USA). cDNA libraries were constructed by the ISU DNA Facility according to the instructions in the Illumina sequencing manual. The Illumina HiSeq 3000 150 paired-end platform was used for sequencing by the DNA Facility at Iowa State University (http://www.dna.iastate.edu/).

Library adapter and low quality nucleotides were trimmed off using Trimmomatic (Bolger et al., 2014). Then, STAR was applied to align the trimmed reads to the reference genome V4.1 of switchgrass (Dobin et al., 2013). The number of reads mapped to each gene was calculated using HTseq-count (Anders et al., 2015). Normalization was done using the Upper Quartile method and gene expression differences were analyzed with package edgeR (Robinson et al., 2010) in the statistical software ‘R’ (Version 3.4.3) (The R FAQ, https://CRAN.R-project.org/doc/FAQ/). Genes are classified as differentially expressed when an absolute log2-fold change value is ≥1 and a false discovery rate is ≤ 0.05 (Benjamini and Hochberg, 1995). Gene Ontology (GO) analysis for DEGs were performed using the Database for Annotation, Visualization and Integrated Discovery (DAVID) (Huang et al., 2009a; Huang et al., 2009b). ReviGO (Supek et al., 2011) and Cytoscape (Shannon et al., 2003) were applied to visualize enriched GOs. Six genes were selected to validate the expression patterns determined by RNA-seq. Primers were listed in **Table S6**.

## Results

### Micropropagation Can Effectively Generate Nonchimeric Mutants From Chimeric Primary (T0) CRISPR/Cas9-Induced Mutants

We micropropagated the primary (T0) *Pvtb1* mutants (52-1 and 35-2) using *in vitro* node culture (**Figure S1**). After about 8 weeks of culture, 48 and 20 plants were successfully regenerated from the primary mutants 35-2 and 52-1, respectively.

To determine the allelic compositions of *Pvtb1a* and *Pvtb1b* genes in 10 randomly selected micropropagated plants including 9 plants generated from primary mutants and 1 plant from the wild-type, sequencing libraries for the *tb1a* amplicons with an insert size of 250 bp and *tb1b* amplicons with an insert size of 288 bp were constructed for each individual plant. For each library, at least 5,000 reads were generated from Illumina HiSeq3000 to determine the sequence of the respective amplicons. Three distinct genotypes were found among the five micropropagated plants (52-1-1, -2, -3, -4, -5) derived from 52-1, while four distinct genotypes were observed among the four micropropagated plants (35-2-1, -2, -3, -4) derived from 35-2, clearly indicating that the two primary *Pvtb1* mutants from which micropropagated plants were obtained are chimeric (**Table 1**).

**Table 1:** Estimation of allelic composition of *Pvtb1* genes in plants regenerated from node culture of the primary mutants 52-1 and 35-2.

Plants name(genotype)	Allelic compositions of *Pvtb1* genes
52-1-1 52-1-4 52-1-5 (Aabb)	Pvtb1a **CCC**CATGGACTTACCGCTTTACC……………………**CCA**CCTCAGCTACCAGCTCGGTA WT/WT 50% Pvtb1a **CCC**CAT-GACTTACCGCTTTACC…………………….**CCA**CCT***T***CAGCTACCAGCTCGGTA −1/+1 50% Pvtb1b **CCC**CAT-GACTTACCGCTTTACC……………………**CCA**CCTCAGCTACCTGCTCGGTA −1/WT 50% Pvtb1b **CTC**CATG–CTCA–GCTTTACC………………………**CCA**CCTC***A***AGCTACCTGCTCGGTA Mix/+1 50%
52-1-2 (Chimeric)	Pvtb1a **CCC**CATGGACTTACCGCTTTACC……………………**CCA**CCTCAGCTACCAGCTCGGTA WT/WT 40% Pvtb1a **CCC**CAT-GACTTACCGCTTTACC…………………….**CCA**CCT***T***CAGCTACCAGCTCGGTA −1/+1 18% Pvtb1a **CCC**CA—–TTACCGCTTTACC…………………………**CCA**CCTCAGCTACCAGCTCGGTA −5/WT 35% Pvtb1a **CCC**CA——TACCGCTTTACC………………………….**CCA**CCTCAGCTACCAGCTCGGTA −6/WT 7% Pvtb1b **CCC**CATGGACTTACCGCTTTACC……………………**CCA**CCTCAGCTACCTGCTCGGTA WT/WT 24% Pvtb1b **CCC**CAT—TTACCGCTTTACC……………………….**CCA**CCTCAGCTACCTGCTCGGTA −4/WT 28% Pvtb1b **CCC**CAT-GACTTACCGCTTTACC…………………….**CCA**CCTCAGCTACCTGCTCGGTA −1/WT 20% Pvtb1b **CTC**CATG–CTCA–GCTTTACC……………………….**CCA**CCT***A***CAGCTACCTGCTCGGTA Mix/+1 17% Pvtb1b **CCC**CA—–TTACCGCTTTACC…………………………**CCA**CCT***T***CAGCTACCTGCTCGGTA −5/+1 11%
52-1-3 (aabb)	Pvtb1a **CCC**CAT-GACTTACCGCTTTACC……………………**CCA**CCT***T***CAGCTACCAGCTCGGTA −1/+1 50% Pvtb1a **CCC**CAT***G***GGACTTACCGCTTTACC……………….**CCA**CCTCAGCTACCAGCTCGGTA +1/WT 50% Pvtb1b **CCC**CAT-GACTTACCGCTTTACC……………………**CCA**CCTCAGCTACCTGCTCGGTA −1/WT 50% Pvtb1b **CTC**CATG–CTCA–GCTTTACC………………………**CCA**CCTC***A***AGCTACCTGCTCGGTA Mix/+1 50%
35-2-1 (Chimeric)	Pvtb1a **CCC**CATGGACTTACCGCTTTACC……………………**CCA**CCTCAGCTACCAGCTCGGTA WT/WT 100% Pvtb1b **CCC**CATGGACTTACCGCTTTACC……………………**CCA**CCTCAGCTACCTGCTCGGTA WT/WT 81% Pvtb1b ————–ACTTACCGCTTTACC………………….**CCA**CCTCAGCTACCTGCTCGGTA −44/WT 13% Pvtb1b **CCC**CATG————————————————–CAGCTACCTGCTCGGTA −128bp 6%
35-2-2 (AABB)	Pvtb1a **CCC**CATGGACTTACCGCTTTACC……………………**CCA**CCTCAGCTACCAGCTCGGTA WT/WT 100% Pvtb1b **CCC**CATGGACTTACCGCTTTACC……………………**CCA**CCTCAGCTACCTGCTCGGTA WT/WT 100%
35-2-3 (Chimeric)	Pvtb1a **CCC**CATGGACTTACCGCTTTACC……………………**CCA**CCTCAGCTACCAGCTCGGTA WT/WT 72% Pvtb1a **CCC**CAT—TTACCGCTTTACC…………………………**CCA**CCTCAGCTACCAGCTCGGTA −4/WT 22% Pvtb1a **CCC**CATGGACTTACCGCTTTACC……………………**CCA**CCT-AGCTACCAGCTCGGTA WT/-1 6% Pvtb1b **CCC**CATGGACTTACCGCTTTACC……………………**CCA**CCTCAGCTACCTGCTCGGTA WT/WT 87% Pvtb1b **CCC**CATG——————————————————CAGCTACCTGCTCGGTA −128bp 7% Pvtb1b **CCC**CATG—TACCGCTTTACC……………………….**CCA**CCTCAGCTACCTGCTCGGTA −4/WT 6%
35-2-4 (Chimeric)	Pvtb1a **CCC**CATGGACTTACCGCTTTACC……………………**CCA**CCTCAGCTACCAGCTCGGTA WT/WT 100% Pvtb1b **CCC**CATGGACTTACCGCTTTACC……………………**CCA**CCTCAGCTACCTGCTCGGTA WT/WT 75% Pvtb1b **CCC**CA—–TTACCGCTTTACC……………………….**CCA**CCTCAGCTACCTGCTCGGTA −5/WT 25%
WT-1 (AABB)	Pvtb1a **CCC**CATGGACTTACCGCTTTACC……………………**CCA**CCTCAGCTACCAGCTCGGTA WT/WT 100% Pvtb1b **CCC**CATGGACTTACCGCTTTACC……………………**CCA**CCTCAGCTACCTGCTCGGTA WT/WT 100%

Representative sequences of Pvtb1 mutations induced by CRISPR/Cas9 with deletions (dashed lines), insertions (italic, bold letters) and substitutions (red letters). Sequences complementary to PAM sequence are in bold. Sequences between two target sites are indicated by black dots. WT-1 is the WT plant derived from the same callus line from which all the primary mutants were obtained and used as a control.

Four nonchimeric, solid mutants, 52-1-1, 52-1-3, 52-1-4, and 52-1-5 were obtained from the T0 mutant 52-1 with 52-1-1, -4, and -5 all having the same genotype, i.e. 50% of WT *Pvtb1a* allele and 50% *Pvtb1a* allele with a G deletion at the first target site and a T insertion at the second target site (**Table 1**), suggesting these plants were likely originated from a single progenitor mutant cell carrying a monoallelic *Pvtb1a* mutation. In the other solid mutant 52-1-3, all alleles of the *Pvtb1a* were mutated with half of the *Pvtb1a* alleles contains a G deletion at the first target site and a T insertion at the second target site, while the remaining half of *Pvtb1a* alleles contains only one change, an insertion of G at the first target site (**Table 1**), suggesting 52-1-3 was likely derived from a single progenitor mutant cell carrying biallelic mutations of *Pvtb1a* gene. These four plants all contain the same biallelic mutations for *Pvtb1b* with no WT alleles (**Table 1**). Fifty percent of the amplicons from each of the four solid mutants contained one C deletion at the first target site, while the remaining half amplicons contained the same mixed mutation (deletions, insertions and substitution) at the first target site and one A insertion at the second target site (**Table 1**), suggesting these four plants were developed from a single progenitor cell or mutant sector carrying biallelic mutations of the *Pvtb1b* gene.

Some of the micropropagated plants remained chimeric following micropropagation. In 52-1-2, four types of *Pvtb1a* amplicons including 3 different mutant alleles accounting for 60% of the reads and 40% WT reads were found (**Table 1**). In 35-2-3, there were three types of *Pvtb1a* amplicons including 28% mutated amplicons and 72% WT (**Table 1**). For *Pvtb1b*, in 52-1-2, 35-2-1, 35-2-3, and 35-2-4, the percentage of mutant reads are 76%, 19%, 13%, and 25%, respectively (**Table 1**). The makeup of the WT and mutant alleles is not proportional to the expected allelic composition for a tetraploid species, strongly indicating these four micropropagated plants (52-1-2, and 35-2-1, -3, -4) remain chimeric mutants comprising a mixture of mutant cells carrying different mutations induced by CRISPR/Cas9. These results indicate the axillary bud contained in the explants used for node culture were likely chimeric.

### Knockout of *Pvtb1* Genes Leads to Increased Tillering in Switchgrass

**Figure 1:**
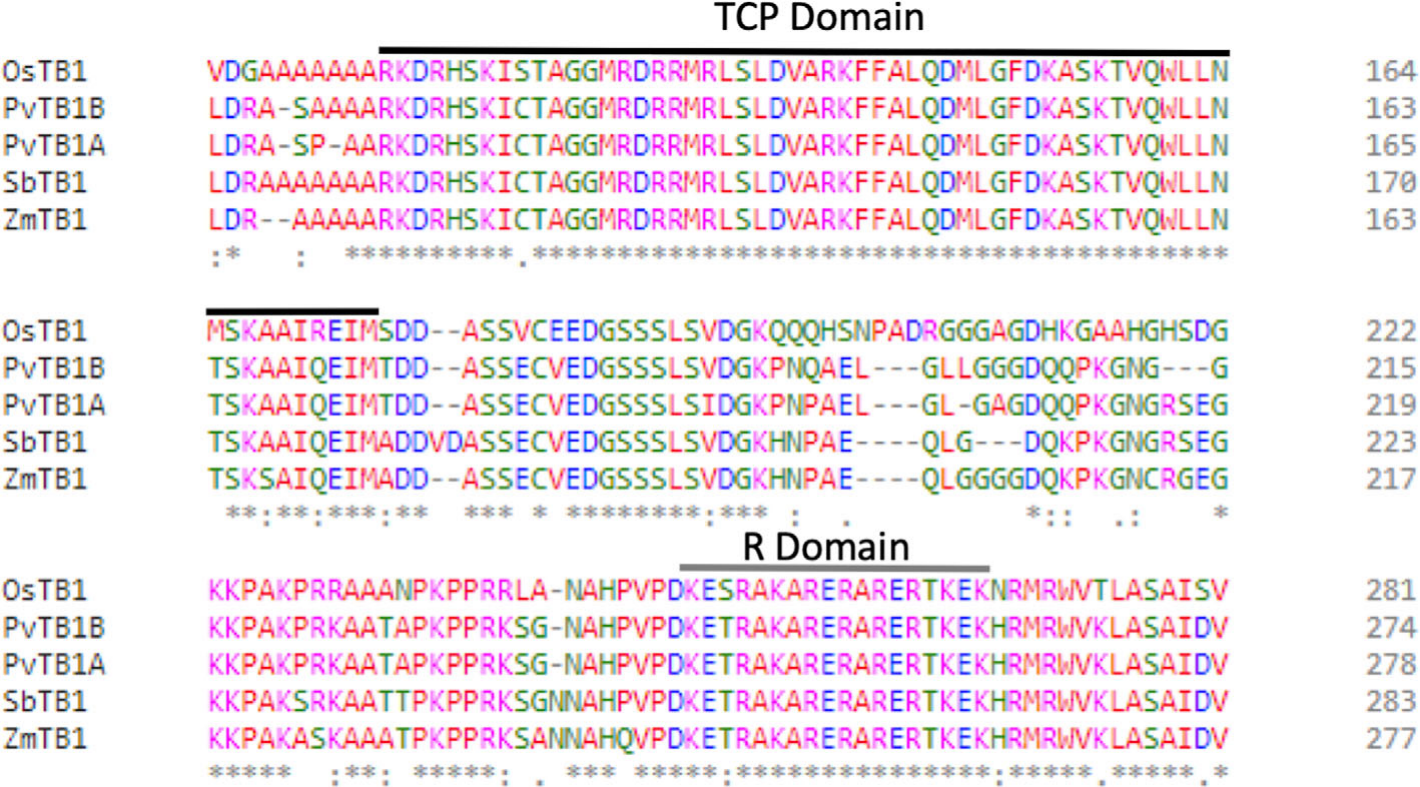
Multiple sequence alignment of Teosinte branched 1 (TB1) proteins from various species. The black line indicates the TCP domain, while the grey line indicates the R domain. Protein names are shown before each sequence.

**Figure 2:**
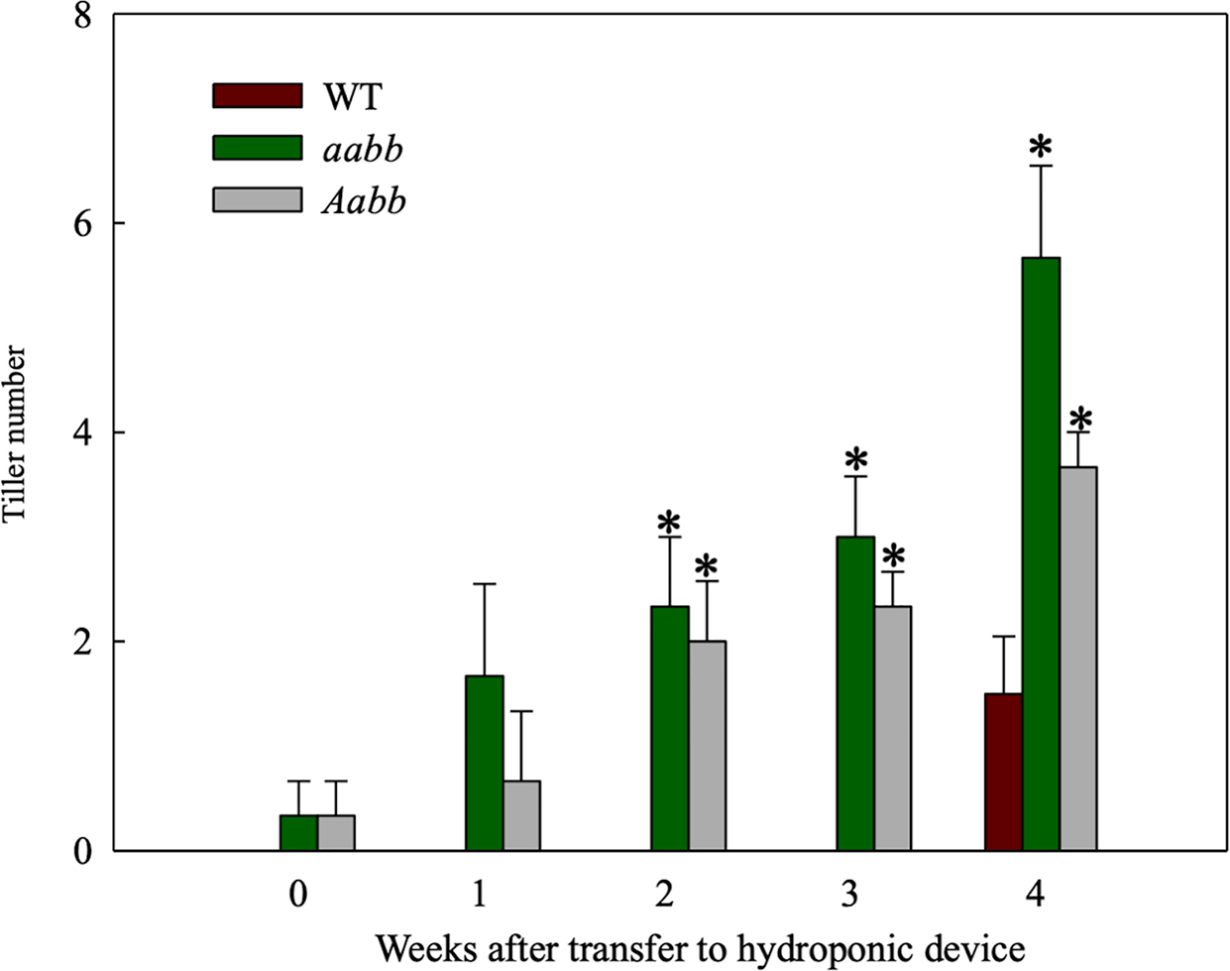
Tiller development in the double biallelic mutant 52-1-3 (*aabb*) and 52-1-1 mutant (monoallelic for *Pvtb1a* and biallelic for *Pvtb1b, Aabb*) and the wild type (WT) at various times after transfer to hydroponic devices. Values are means ± s.d. (mutants, n = 3 plants; WT, n = 6 plants). * indicated significance differences between mutants and WT at P < 0.05.

Based on the most recent annotated switchgrass genome in Phytozome (Goodstein et al., 2012) (https://phytozome.jgi.doe.gov/pz/portal.html), two *tb1-like* genes, *Pvtb1a* (Pavir.Ia00838/Pavir.9NG142700) and *Pvtb1b* (Pavir.Ib04362/Pavir.9KG031700), were isolated from switchgrass using gene-specific primers. These two *Pvtb1* genes reside on homeologous chromosomes 9N and 9K, thus are likely homeologous to each other. Amino acid sequence alignment of *Pvtb1* gene products and other *tb1* orthologs of related species showed that both PvTB1 proteins have the conserved TCP domain and R domain (**Figure 1**). To determine the function of *Pvtb1* genes, tiller numbers of the *Pvtb1a-Pvtb1b* double biallelic mutants (*aabb*), *Pvtb1a* monoallelic and *Pvtb1b* biallelic mutant plants (*Aabb*) and WT (AABB) plants were compared. Because switchgrass tillers develop from nodes near or below soil surface, we grew plants in a hydroponic system, which allows close monitoring of tiller and root growth without the destructive excavation of the underground parts of these plants. As shown in **Figure 2**, the first primary tiller of WT plants did not develop until the end of the fourth week following establishment in the hydroponic system, while most of the mutant plants developed their first primary tiller by the end of the first week. After 4 weeks, the average tiller number of *aabb* mutants is 5.7 which is about two times the tiller number of WT plants, while the average tiller number of *Aabb* mutants is 3.7 which is about 1.5 times the tiller number of WT plants.

**Figure 3:**
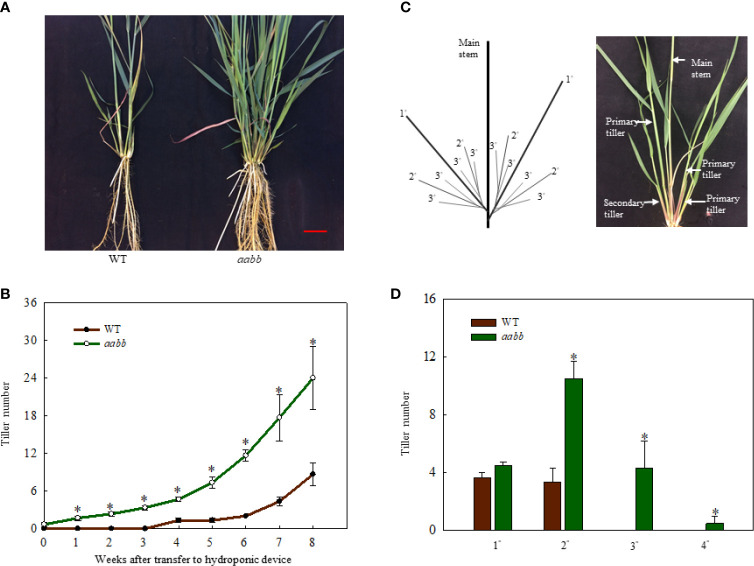
Phenotypic characterization of the *Pvtb1a-Pvtb1b* biallelic mutant (52-1-3, *aabb*) and the WT. **(A)** Phenotype of the *Pvtb1a-Pvtb1b* biallelic mutant and the WT after 8 weeks of growth in a hydroponic device. Bar = 3 cm. **(B)** Weekly changes in tiller numbers in the 52-1-3 mutant and the WT after transfer to a hydroponic device. **(C)** Schematic diagram for tiller ordering in switchgrass. 1° denotes primary tillers, 2° denotes secondary tillers, 3° denotes tertiary tillers. **(D)** Number of tillers of different orders in the aabb mutant and the wild type (WT) after 8 weeks of growth in hydroponic devices. Values are means ± s.d. [**(B)**, n = 3 plants; **(D)**, n = 6 plants]. * indicated significant differences at *P* < 0.05.

**Figure 4:**
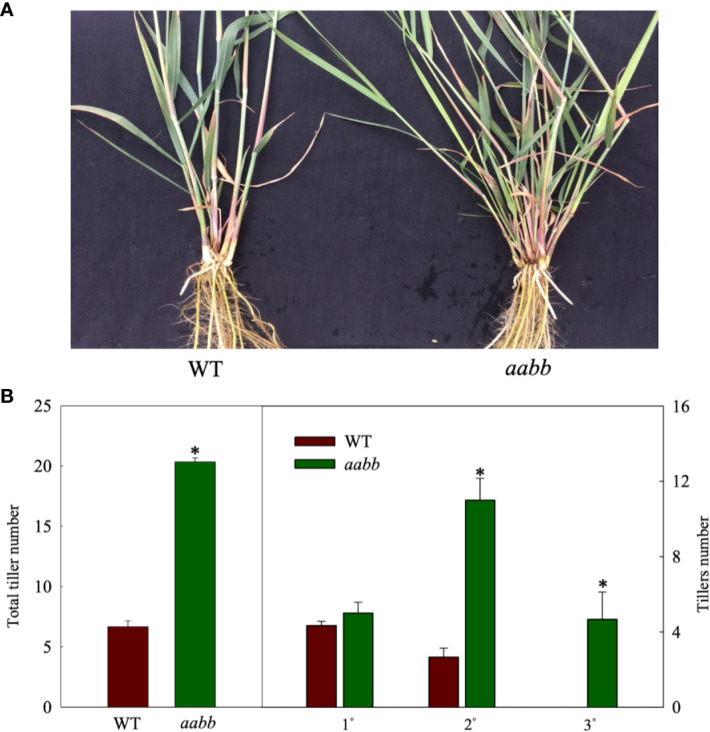
Phenotypic characterization of tiller production with primary tillers developed from main stems of the *Pvtb1a-Pvtb1b* double biallelic mutant (52-1-3, *aabb*) as starting materials and cultured in a hydroponic devise for 8 weeks. **(A)** Visual appearance of the wild type (WT) (left) and the mutant (right) after 8 weeks of culture. **(B)** Total number of tillers and tiller number for each class of tillers after 8 weeks of culture. 1° denotes primary tillers, 2° denotes secondary tillers, 3° denotes tertiary tillers. Values are means ± s.d. (n = 3 plants). * indicated significant differences at *P* < 0.05.

To further investigate where the increased tiller originated from mutants, development of tillers of different orders in the *aabb* mutant plants and WT plants were closely monitored for 8 weeks in a separate hydroponic experiment. As shown in **Figure 3A**, the *pvtb1a-pvtb1b* (aabb) mutant plants had significantly more tillers than WT plants. The change of tiller numbers over the course of 8 weeks show the tiller numbers of mutant plants are significantly higher than WT plants at all weekly sampling times (**Figure 3B**). The WT plants did not generate new tillers until the end of the fourth week after establishment in the hydroponic system, while the mutant plants started to produce new tillers at the end of the first week, which is similar to the results from the shorter-term study described above. However, the *pvtb1a-pvtb1b* mutant plants had no significant effect on the number of primary tillers (1°) compared with WT plants (**Figures 3C, D**), which suggested that *Pvtb1* genes do not regulate the formation of primary axillary buds in the main stem, and the significant increase in secondary (2°), tertiary (3°), or quaternary (4°) tillers in the mutant plants is the result of hastened outgrowth of primary axillary buds. To further confirm the effect of *Pvtb1* on the outgrowth of axillary buds and eliminate possible effect of physiological conditions of the starting materials, primary tillers developed from the *pvtb1a-pvtb1b* double biallelic mutant plants and the WT plants were grown as starting material in a separate hydroponic study. The total number of new tillers developed from the primary tillers of the mutant plants was three-fold of that produced by the primary tillers of the WT plants (**Figures 4A, B**). Similarly, plants established from the primary tillers of the *pvtb1a-pvtb1b* mutant had similar number of the first order tillers (1°) to that of the WT, but had greatly increased number of secondary (2°) and tertiary tillers (3°) than those from the primary tillers of the WT. These results strongly suggest that, regardless of the origin of the starting materials, the *pvtb1a-pvtb1b* mutant plants consistently promote axillary bud outgrowth, increasing tiller number. Aerial tillers, however, were not observed in either the mutant plants or the WT plants, suggesting that *Pvtb1* genes did not affect the outgrowth of the upper axillary buds. Interestingly, while the first axillary bud (the lowest in the stem base) in the WT was generally dormant, it was frequently elongated to eventually become a tiller in the *pvtb1a-pvtb1b* mutant (**Figure S2**). These results further demonstrate that *Pvtb1* genes affect the outgrowth of the axillary bud rather than the formation of new axillary buds.

### *Pvtb1* Knock-out Mutant Plants Exhibit Increased Root Production and Biomass Yield

**Figure 5:**
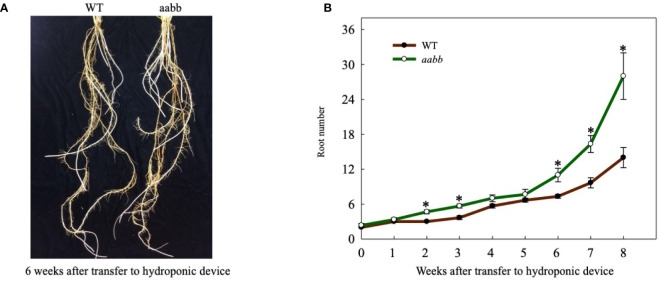
Phenotypic characterization of root development for the *Pvtb1a-Pvtb1b* biallelic mutant (52-1-3, *aabb*) and the wild type (WT). **(A)** Representative plants of WT and a mutant (*aabb*) with increased root numbers are shown. **(B)** Weekly changes in root number in the *Pvtb1a-Pvtb1b* double biallelic mutant (52-1-3, *aabb*) and the WT after transfer to hydroponic devices. Values are means ± s.d. (n = 3 plants). * indicated significant differences at *P* < 0.05.

The average root number in mutant plants was similar to that of the WT plants at the beginning of the hydroponic culture (**Figure 5**). Starting from the second week, the difference between the average root number in mutant plants and WT plants started to increase as the experiment progressed (**Figure 5**). At the end of the experiment, the average root number of the mutant plants was 28, twice the average root number of the WT plants (**Figure 5**). Hence, disrupting *Pvtb1* gene function in switchgrass enhances root production.

To eliminate the potential unintended effect of the hydroponic growth environment on growth and development, we also evaluated tiller number, stem diameter, plant height, fresh weight and dry weight of *pvtb1a-pvtb1b* mutant plants and WT plants grown in the soil. Similar to the hydroponic experiments, *pvtb1a-pvtb1b* mutant plants produced significantly more tillers (2.53-fold) compared with the WT plants (**Table 2**). Plant height of the mutant plants was similar to that of the WT, while stem diameter of the mutant plants was 13.6% smaller than that of the WT (**Table 2**). The fresh and dry biomass of the mutant plants increased 29.6% and 15.5% over these of the WT plants, respectively, suggesting the enhanced numbers of tillers of mutants were more than sufficient to compensate for the loss in biomass from decreased stem diameter (**Table 2**).

**Table 2:** Phenotype of the wild-type (WT) and the micropropagated biallelic mutant plants, after 12 weeks of growth in pots filled with soil.

Genotype	Tiller number	Stem diameter (mm)	Plant height (m)	Fresh weight (g plant^-1^)	Dry weight (g plant^-1^)
AABB (WT)	22.4 ± 1.44^b^	4.56 ± 0.13^a^	1.67 ± 0.11^a^	34.5 ± 1.30^b^	10.3 ± 0.98^a^
*aabb* (52-1-3)	56.6 ± 4.31^a^	3.94 ± 0.14^b^	1.45 ± 0.07^a^	44.7 ± 4.13^a^	11.9 ± 0.71^a^

Values are means ± s.d. (n = 5 plants). Different letters at the same column indicate significant differences at P < 0.05 level.

### CRISPR/Cas9-Induced Mutations Are Transmitted to Progeny

Only transgene-free BC1 plants, derived from crossing the T0 mutants as maternal parents with a compatible WT plant, were examined to avoid complications that could have resulted from the continuing action of the CRISPR/Cas9 transgene. Among the 30 progeny of the primary mutant 52-1, 12 were transgene-free with ten of them carrying mutations. Meanwhile three of six progeny of the cross between mutant 35-1 and a WT plant carried no transgenes and of these, only one carried mutations (**Table 3**). Additionally, 18 of 20 progeny from the mutant 35-2 were transgene-free (**Table 3**), but only two carried mutation.

**Table 3:** Transgene-free BC1 mutants obtained from crossing each of the primary mutants with genetically compatible wild-type plants.

T0 Mutant	# Progeny analyzed^†^	# of Cas9 negative^‡^	Cas9 negative			
			Monoallelic *Pvtb1a-Pvtb1b* mutant	Monoallelic *Pvtb1b* mutant	Monoallelic *Pvtb1a* mutant	Nonmutant
52-1	30	12	5	5	0	2
35-2	20	18	0	0	2	16
35-1	6	3	1	0	0	2

^†^BC1 seeds collected from each of the primary mutants were germinated and allowed to grow for a month.

^‡^The presence of the transgene was analyzed using PCR with gRNA/Cas9-specific primers (**Supplementary Table S6**).

The *Pvtb1* mutations observed in all three primary mutants were also observed in their progeny, indicating a stable transmission of the original mutations to the progeny (**Table S1**). Seven BC1 plants of the 52-1 had both *Pvtb1* genes mutated (**Table S1**), while the other five plants, in BC1 generation, only contained mutations of *Pvtb1b* (**Table S1**). All mutations in these BC1 plants were present in the primary mutant 52-1 except for the 52-1-BC1-24, which has a mutated *Pvtb1a* allele carrying a 128 bp deletion which was not observed in the primary 52-1 mutant (**Table S1**). Two monoallelic *Pvtb1a* mutants (35-2-BC1-1 and -6) were obtained from the progeny of the 35-2 (**Table 3**). Both carried identical mutations observed in the primary mutant 35-2 (**Table S1**). Furthermore, one doubly monoallelic *Pvtb1a-tb1b* mutant with big deletions for both *tb1* genes was found in the progeny of 35-1 (**Table S1**).

To test if mutant progeny still retains the phenotypic effects of *Pvtb1* mutations, tiller numbers of *Pvtb1b* monoallelic mutants (genotype AABb with A represents the WT and a represents the mutant allele of *Pvtb1a* and B represents the WT and b represents the mutant allele of *Pvtb1b*), and *Pvtb1a-Pvtb1b* doubly monoallelic mutant plants (genotype AaBb) in the BC1 generation were investigated. Tiller numbers of doubly *Pvtb1a-Pvtb1b* monoallelic mutants and single *Pvtb1b* monoallelic mutants were significantly higher than the siblings carrying no mutations (null segregants), suggesting that *Pvtb1* genes act in dosage-dependent manner. The average percentage increase in tiller number was 45% for doubly *Pvtb1a-Pvtb1b* monoallelic mutant plants and 30% for the *Pvtb1b* monoallelic mutant plants (**Table 4**).

**Table 4:** The average tiller number of transgene-free BC1 mutants and that of the siblings carrying no mutations (null segregants) and the percentage increase of tiller number for mutants over nonmutant siblings.

Genotype	N	Average tiller number	% increase of tiller number in mutant over the WT
AABb (mutant)	4	26.0 ± 4.7^b^	30 ± 23
AaBb (mutant)	5	29.2 ± 4.4^b^	45 ± 20
AABB (WT)	6	20.0 ± 1.4^a^	0 ± 10

All data are shown as mean ± standard deviation. A and a represent the wild-type and mutant allele of Pvtb1a gene, respectively whereas B and b represent the wild-type and the mutant allele of the Pvtb1b gene, respectively. The percentage increase of tiller number for each genotype was calculated by comparing the mutant plants with the wild-type plants at the same developmental stage using the one-tailed Student’s t-test. Different letters at the same column indicate significant differences at P < 0.05 level.

### mRNA-Seq of *Pvtb1* Mutants Suggests *Pvtb1* Genes Are Involved in Multiple Pathways That Regulate Tillering in Switchgrass

Although the primary mutant 52-1 was later found to be chimeric, its enhanced tiller production (Liu et al., 2018) with 30%–50% of the *tb1a* alleles and 61%–94% of the *tb1b* alleles mutated are sufficient to be treated as a knockdown mutant of *Pvtb1* gene (**Figure S3**). Using a generalized linear model with the R package edgeR (Robinson et al., 2010), 831 genes showed significantly differential expression between the WT and 52-1 when the false discovery rate (FDR) was controlled at 0.05 (Benjamini and Hochberg, 1995) (**Table S2**). Among them, 364 genes were significantly upregulated while 467 genes showed significantly down regulation in the mutant 52-1 (**Figure S4**). To validate the RNA-seq data, qRT-PCR was performed on 4 differentially expressed genes and 2 genes with similar expression level between the WT and 52-1. As shown in **Figure S5**, the expression trends of these 6 genes observed in qRT-PCR and RNA-seq results were similar in WT_vs_52-1 and a high expression correlation was observed between fold changes of 6 genes in RNA-seq and qPCR experiments using linear regression analysis (R^2^ = 0.871, *p*=0.0065), indicating the results of RNA-seq are reliable.

**Figure 6:**
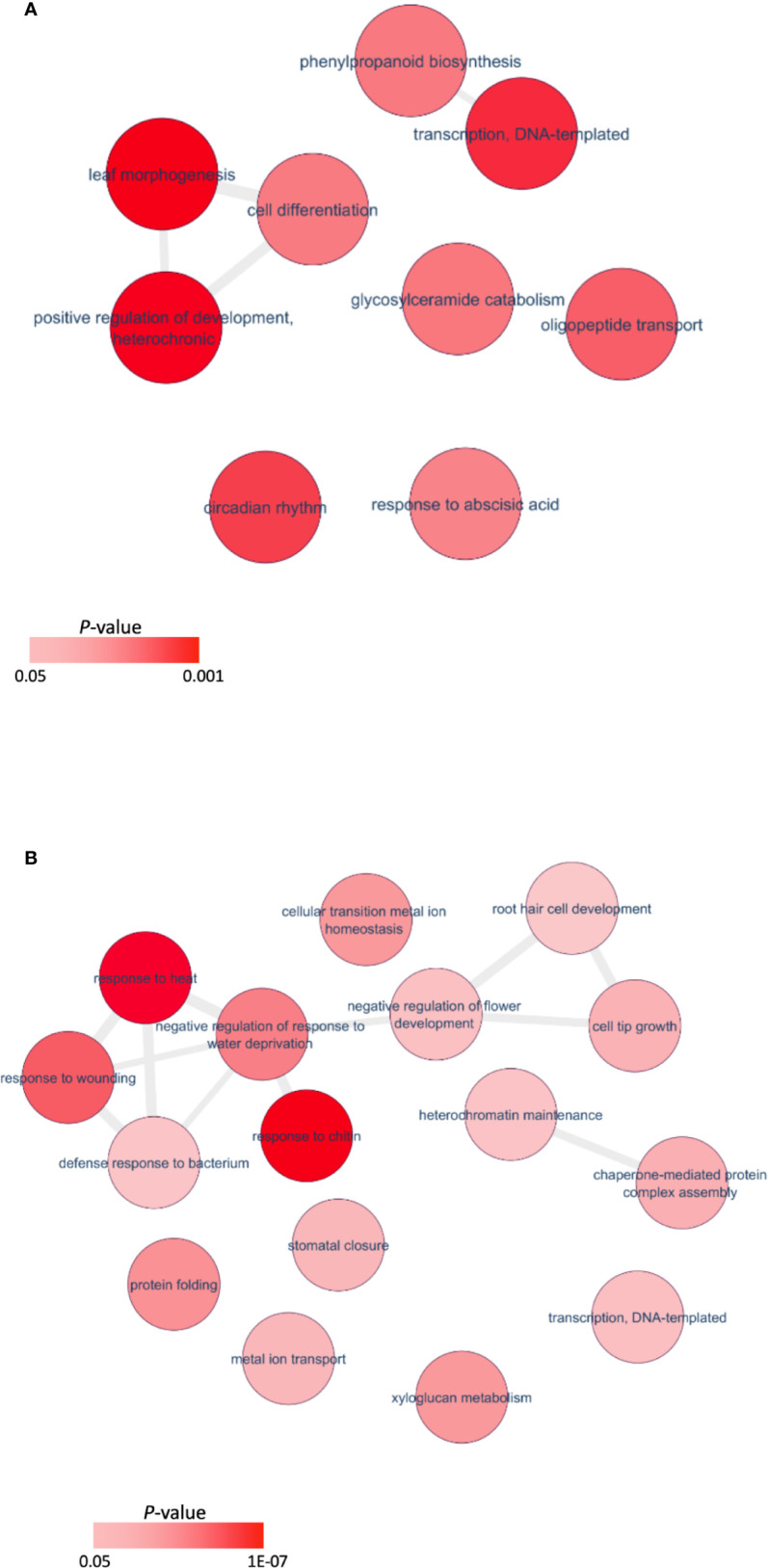
Enriched Gene Ontology (GO) terms and differentially expressed genes of the *Pvtb1* genes knockdown mutant. **(A)** Gene set enrichment analysis of upregulated genes; **(B)** Gene set enrichment analysis of downregulated genes. Each red circle represents a GO term. Two color bars indicate *P-value* ranging from 0.05 to 0.001 and 1.0 × 10^−7^, respectively.

**Figure 7:**
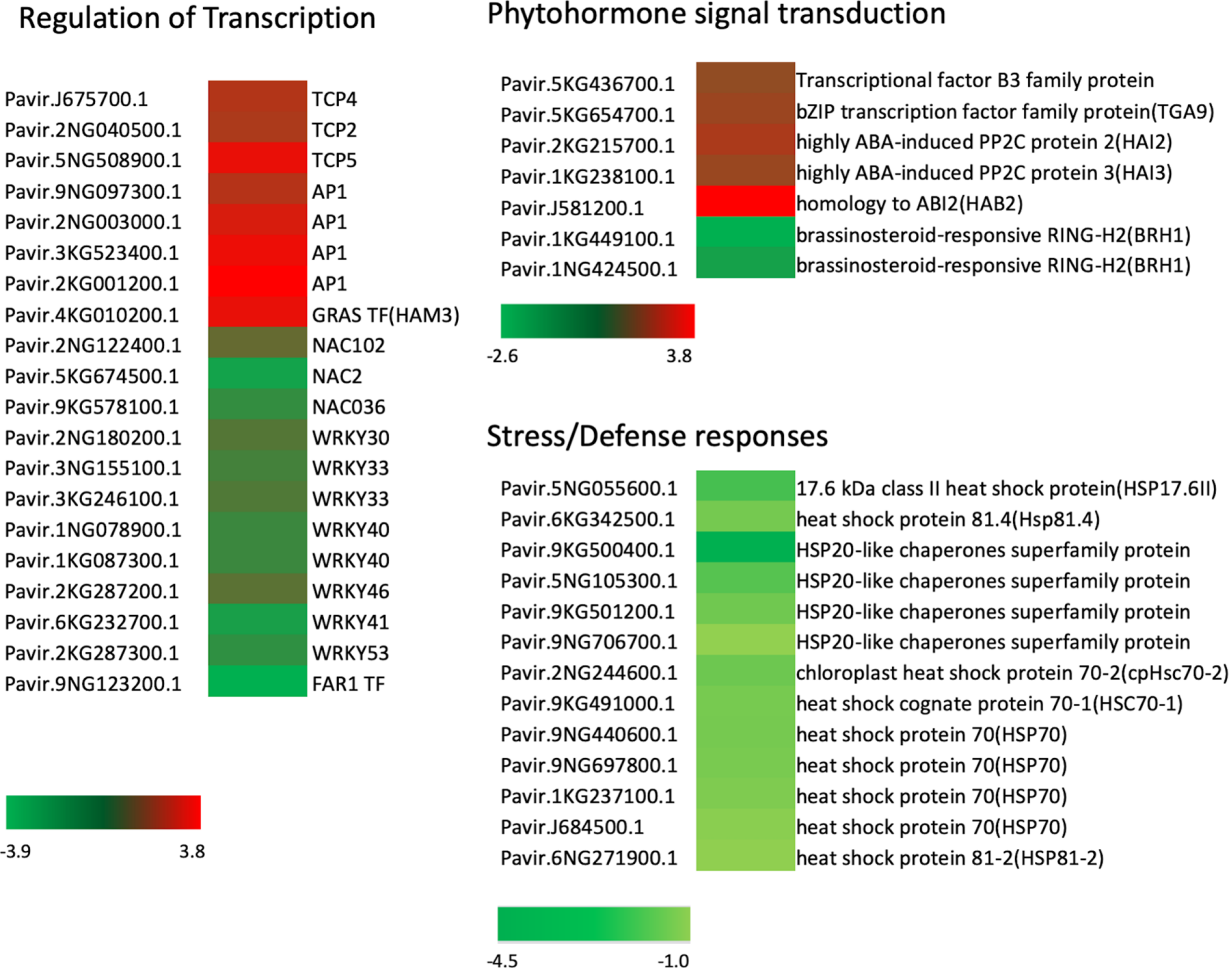
Expression patterns of representative differentially expressed genes (DEGs) involved in transcription regulation, phytohormone signal transduction and stress/defense responses previously characterized in Arabidopsis. Three color bars represent the range of fold change for DEGs, where red indicates upregulation while green indicates downregulation.

Orthologous Arabidopsis genes for these DEGs were subjected to Gene Ontology (GO) analysis using the Database for Annotation, Visualization and Integrated Discovery (DAVID) (Huang et al., 2009a; Huang et al., 2009b). Regarding the biological process, 27 terms (P<0.05, **Table S4**) were enriched for downregulated genes in the mutant, and 12 terms (P<0.05, **Table S3**) for the upregulated genes. A major GO term for these DEGs was DNA-templated transcription (GO: 0006351) (**Figures 6A, B**). Among the genes enriched in this GO term, transcript levels for 34 genes increased significantly in the *Pvtb1* gene knockdown mutant 52-1 while the remaining 43 genes were downregulated (**Table S5**). The majority of the genes in this GO term encode transcription factors (TFs). For instance, genes for 3 TCP family TFs (TCP2, 4, and 5) and 4 MADS-box family TFs (AP1) were upregulated in the mutant 52-1, while genes for 8 WRKY family TFs (WRKY30, 33, 40, 41, 46, and 53) and 3 NAC family TFs (NAC02, 036, and 102) were down- regulated. GO terms associated with cell differentiation and positive regulation of development were also enriched among the upregulated genes in the mutant. Other GOs enriched in both up- and downregulated genes were involved in response to various phytohormones suggesting *Pvtb1* genes are, as in other species involved in various hormonal signaling pathways in regulating tillering in switchgrass (**Tables S3****,** **S4**). For example, BRH1, a brassinosteroid-responsive RING-H2 gene, was downregulated in the *Pvtb1* gene knockdown mutant (**Figure 7**). In contrast, genes for auxin-responsive factor and ABA-induced proteins were both upregulated in the mutant (**Figure 7**). In addition, GO terms involved in negative regulation of flower development and heterochromatin maintenance were overrepresented among the downregulated genes (**Figure 6B**). For example, Arabidopsis DRD1 and EDM2 both regulate DNA methylation patterns (Cho et al., 2016; Duan et al., 2017) and the orthologs of these two were downregulated in the present study. Hence, we speculate that epigenetic modification plays a role in *Pvtb1*-mediated regulation in tillering in switchgrass. Intriguingly, GOs associated with stress/defense responses were significantly enriched in downregulated genes, indicating *Pvtb1* genes might function in maintaining homeostasis in the regulation of growth and defense (**Figures 6B**, **7**). These data suggest the gene regulatory network of tillering in switchgrass is complex and *Pvtb1* genes are important hubs in this network.

## Discussion

CRISPR/Cas9-based genome editing tool affords new possibilities for reverse genetics-based studies in switchgrass (Liu et al., 2018). In the present study, through the node culture of chimeric primary mutants induced by the CRISPR/Cas9 system, we successfully obtained a nonchimeric biallelic *Pvtb1a-Pvtb1b* mutant, and two nonchimeric mutants carrying biallelic *Pvtb1b* mutations and one *Pvtb1a* mutated allele. These results demonstrate that micropropagation is an effective way of isolating mutant sectors from chimeric mutants in switchgrass and obtaining nonchimeric mutants. Solid nonchimeric mutants were isolated from the chimeric primary mutant, 52-1. Among five plants regenerated from nodes culture of 52-1, four plants are nonchimeric mutants, suggesting the primary mutant plant 52-1 is composed primarily of mutant cells. However, for the primary mutant 35-2, none of the regenerated plants are nonchimeric mutants, indicating this plant, or regions where axillary buds arise are composed primarily of nonmutant cells. Overall, we demonstrated that nonchimeric mutants can be successfully isolated from chimeric mutants using micropropagation, but the chimeric pattern of chimeric mutants is a determining factor on the efficiency of isolating nonchimeric mutants, and varies greatly between mutants. Strong genetic-incompatibility of switchgrass is an obstacle to generate homozygous mutants. To set seeds carrying homozygous mutations, individual mutants used for crossing must have different alleles of S and Z genes (Martinez-Reyna and Vogel, 2002), which is difficult to determine because the molecular basis of the self-incompatibility in switchgrass has not been studied. Also, it takes over 6 months for switchgrass seedlings to reach reproductive stage (Hopkins et al., 1995; VanEsbroeck et al., 1997) for crossing. Given all these obstacles, the successful purification of chimeric mutants using micropropagation clearly has advantages. In addition, nonchimeric homozygous mutants can be obtained from T0 chimeric mutants within one month of using the micropropagation method, which is time-saving compared to traditional breeding method.

Switchgrass cv. Alamo is an allotetraploid with two homeologous subgenomes, but detailed information about chromosome pairing, whole or partial genome duplications, and allelic diversity of specific genes is lacking (Missaoui et al., 2005; Okada et al., 2010). With limited information gained by sequencing clones of PCR amplicons spanning the target regions, the nature of mutants was not resolved unequivocally in our previous study (Liu et al., 2018). Using the Next Generation Sequencing (NGS) technology, genotypes of micropropagated mutants were fully characterized. The two primary mutants, 52-1 and 35-2 were both revealed by NGS to be chimeric mutants, which were likely the results of continuous action of CRISPR/Cas9 in somatic cells. Indeed, chimeric mutations induced by CRISPR/Cas9 have been reported in different species (Feng et al., 2014; Pan et al., 2017). For instance, Pan et al. (2017) showed that 63.9% T0 transgenic plants carried chimeric mutations in tomato (*Solanum lycopersicum* L.). In rice, the segregation ratio of CRISPR/Cas9-induced mutations in BC1 generation did not follow the expected segregation ratio, indicating the chimeric mutations in T0 plants (Xu et al., 2015).

Transgene-free mutants were successfully obtained by crossing primary mutant plants with WT plants. The majority of the mutations observed in progeny were identical to the mutations in the primary mutants. For example, BC1 progeny of the 52-1 carried the *Pvtb1b* mutations that were present in all five tillers of the primary mutant 52-1. These results demonstrate mutations induced by the CRISPR/Cas9 in T0 mutants were stably transmitted to the BC1 generation without alteration. However, a mutation (128bp deletion) of *Pvtb1a*, not detected in the primary mutant 52-1, was observed in the BC1 transgene-free mutant plant 52-1-BC1-24. This deletion likely escaped detection in T0 plants due to the chimeric nature. The similar phenomenon has also been observed in maize (Lee et al., 2019).

*Pvtb1* genes are closely related to the *tb1* genes in other monocots. The *Pvtb1a-Pvtb1b* double biallelic mutants produced significantly higher tiller number than the WT plants under both hydroponic and soil culture conditions. Results from the hydroponic experiments in which starting plant materials were primary tillers validated the observed differences between the mutants and the WT plants were the result of genetic alteration instead of the existing physiological difference between the WT and the mutant. Our results strongly indicate *Pvtb1* genes play an important role in regulating tillering in switchgrass. There was no significant difference in the number of primary tillers between the WT and 52-1-3, suggesting the function of *Pvtb1* genes in switchgrass is only to regulate the rate of outgrowth of axillary buds that are destined to become tillers with one possible exception, i.e., the release of the outgrowth of the bud at the lowest node (**Figure S2**). This is similar to orthologs of *tb1* in other species, reflecting the functional conservation of *tb1* genes across species (Takeda et al., 2003; Kebrom et al., 2006; Aguilar-Martinez et al., 2007; Braun et al., 2012).

The *Pvtb1a-Pvtb1b* double biallelic mutant plants also produced more roots compared with WT plants. This result is consistent with Gaudin et al. (2014) who reports a decrease in *tb1* function in maize resulted in a larger root system. The increased root growth and development is likely the indirect result of increased tiller production, as each tiller normally develops its own adventitious roots. Increased tiller number has the potential to increase biomass yield in switchgrass (Chuck et al., 2011; Fu et al., 2012). The *Pvtb1a-Pvtb1b* mutants produced 29.6% and 15.5% more fresh and dry biomass, respectively. Although the difference on dry biomass between the wild-type and mutant plants is not statistically significant, this may change if mutant plants with more tillers and increased root mass are grown in the field where more resources are available.

In maize, studies have shown that *tb1* regulates branching in a dosage-dependent manner (Doebley et al., 1995; Hubbard et al., 2002). Maize heterozygous *tb1* mutants had slightly more tillers than WT plants, while homozygous *tb1* mutants produced more tillers than heterozygous *tb1* mutants (Doebley et al., 1995). In this study, we noticed the tiller number in monoallelic heterozygous *Pvtb1b* mutants (AABb) was significantly higher than WT plants, suggesting that *Pvtb1b* functions in a dosage-dependent manner. In addition, due to the high amino acid sequence identities between PvTB1A and PvTB1B, it is reasonable to expect they regulate tillering of switchgrass redundantly or additively. However, comparing monoallelic *Pvtb1b* mutant plants (AABb) with the doubly monoallelic *Pvtb1a-Pvtb1b* mutant plants (AaBb) did not show significant differences on tiller numbers, suggesting there is no significant additive effect between *Pvtb1a* and *Pvtb1b*. Therefore, *Pvtb1a* might have a minor effect on tillering in switchgrass. This is similar to Arabidopsis in which only BRC1 regulates branching, despite both BRC1 and BRC2 have the conserved TCP and R domains (Aguilar-Martinez et al., 2007; Gonzalez-Grandio et al., 2013; Seale et al., 2017).

To have a better understanding of the functions of *Pvtb1* genes, we examined global transcriptional changes caused by the downregulation of *Pvtb1* genes. Increased expression level of genes for TCP TFs that are associated with cell differentiation and positive regulation of development in the mutant suggested that *Pvtb1* genes inhibit the tiller production through deactivating cell differentiation. In addition, *HAIRY MERISTEM 3* (*HAM3*), the gene for GRAS family TF that interacts with WUSCHEL (WUS) TF to promote shoot meristem development (Zhou et al., 2015), was upregulated in the mutant, suggesting increased shoot stem cell proliferation in the *Pvtb1* gene knockdown mutant.

Our transcriptomic analysis results suggest PvTB1a and PvTB1b regulate tillering in switchgrass by interacting with complex hormonal signaling pathways. Six cytochrome P450 genes were up regulated in the mutant (**Table S5**). The members of cytochrome P450 family catalyze the biosynthesis of several phytohormones including auxin, brassinosteroids, and strigolactones which regulate branching across various plants species (Zhao, 2008; Kebrom et al., 2013). In addition, increased expression of ABA-responsive genes, *PROTEIN PHOSPHATASE 2C* (*PP2C*) genes, was observed in the mutant. These results suggest that *Pvtb1* genes regulate bud development by modulating phytohormone biosynthesis and signaling. In Arabidopsis and maize, it has been shown TB1/BRC1 promotes ABA accumulation and the expression of ABA response factors to inhibit bud outgrowth (Gonzalez-Grandio et al., 2013; Yao and Finlayson, 2015; Gonzalez-Grandio et al., 2017; Holalu and Finlayson, 2017). Although it is well-known that *TB1*/*BRC1* are involved in hormonal signaling pathways in different plant species, these regulation pathways are not conserved across various species (Kebrom et al., 2013). For instance, cytokinins repress the expression of *TB1* in rice, while they act in a pathway independent of BRC1 in Arabidopsis (Aguilar-Martinez et al., 2007; Minakuchi et al., 2010). The timing of hormonal signaling in tillering has not been decided in switchgrass. Hence, more studies are needed to understand how the hormonal signals are involved in the *Pvtb1*-mediated regulation of branching.

Altered expression of genes in response to red or far-red light in the mutant suggest that *Pvtb1* genes integrate the light signal to regulate tillering in switchgrass (**Figure 7**). *FAR-RED-IMPAIRED RESPONSE1* (*FAR1*)-related sequence (FRS) family of transcription factors regulate plant growth and development in response to far-red light in Arabidopsis (Wang and Wang, 2015). The homolog of *FAR1*, *FAR-RED ELONGATED HYPOCOTYLS3* (*FHY3*) promotes shoot branching in Arabidopsis (Stirnberg et al., 2012). Further, *FAR1*/*FHY3* promotes *FHY1*/*FHL* gene expression to facilitate phyA nuclear accumulation under far-red light condition. The downregulation of FRS TF genes in the switchgrass mutant suggested that *Pvtb1* genes may be involved in promotion of phyA nuclear accumulation to inhibit the axillary bud initiation or outgrowth. Additionally, knockdown of *Pvtb1* genes increased the expression level of the *Phytochrome interacting factor 4* (*PIF4*) gene that has been shown to regulate expression of genes involved in cell expansion (Huq and Quail, 2002). Because PIF4 is a TF regulated by phyB-mediated signaling, its activity is regulated by the red light signal (Xu, 2018). These results suggest *Pvtb1* genes regulate tillering through light signaling pathways. It has been reported that *tb1* genes inhibit bud outgrowth in the process of shade-avoidance-syndrome (SAS) (Kebrom et al., 2013). In Arabidopsis and sorghum, the expression levels of *BRC1* and *SbTB1* were both upregulated under shade (Kebrom et al., 2006; Kebrom et al., 2010; Gonzalez-Grandio et al., 2013). Therefore, *Pvtb1* genes might also sense the low R:FR ratio to inhibit bud outgrowth in switchgrass.

Several genes associated with stress/defense responses were significantly downregulated in the mutant. For example, 13 genes for Heat-shock proteins (Hsps)/chaperones which assist in protein refolding under stress conditions (Wang et al., 2004) were downregulated in the mutant. It has been shown overexpression of alfalfa (*Medicago sativa* L.) *MsHSP70* gene could enhance Arabidopsis drought and heat stress tolerance (Li et al., 2017). Soybean (*Glycine max* (L.) Merril) Hsp 90 family members respond differentially to abiotic stresses and reduce the damage caused by abiotic stresses in Arabidopsis (Xu et al., 2013). Hence, the downregulation of Hsp genes in the switchgrass mutant suggest hastened outgrowth of axillary buds of the mutant might trigger increased expression of stress/defense responsive genes. Further, the expression levels of several WRKY TF genes responsive to chitin elicitation functioning in plant defense to fungal pathogens (Libault et al., 2007) also decreased in the mutant. In Arabidopsis, WRKY TFs are necessary for resistance to pathogen infection (Zheng et al., 2006) or resistance to abiotic stresses (Chen et al., 2010). It is well-known tolerance-growth trade-offs occur in plants under the low-resource conditions (Koziol et al., 2012; Bristiel et al., 2018). It has been shown that TB1 may influences sucrose levels and energy balance within dormancy buds in maize (Dong et al., 2019). Exploration of the mechanism of PvTB1s controlling energy balance in switchgrass would provide valuable information for the improvement of switchgrass for biomass production and development of enhanced stress-tolerant cultivars.

## Conclusions

We successfully isolated mutated segments from chimeric mutants using micropropagation. This method overcomes the difficulties of obtaining nonchimeric mutants in self-incompatible species. Further, transgene-free mutants were obtained in this research, which provided valuable germplasm for switchgrass genetic research and breeding. More importantly, we proved the stable transmission of mutations induced by the CRISPR/Cas9 system in switchgrass. We propose that *Pvtb1* genes negatively regulates tillering in switchgrass. RNA-seq analysis revealed a complex regulatory network potentially regulating tillering in switchgrass and provided some clues to the pathways of *Pvtb1* genes.

## Data Availability Statement

The datasets presented in this study can be found in online repositories: The repository name is Sequence Read Archive (SRA). The project name is' Transcriptome sequencing of axillary buds of switchgrass Pvtb1 mutant and WT plant' The accession number is **PRJNA660286**. To access the data, please use the link: https://www.ncbi.nlm.nih.gov/Traces/study/?acc=PRJNA660286.

## Author Contributions

S-zF and YL conceived the original research plans. YL conducted the experiments on gene editing, micropropagation, and sequencing and analyzed relevant data. YB provided technical assistance to YL. WW conducted the hydroponic experiment. CC provided technical assistance to WW. YL and S-zF wrote the article with contributions from all authors. S-zF agrees to serve as the author responsible for contact and ensures communication.

## Funding

This work was partially supported by the National Institute of Food and Agriculture of the US Department of Agriculture (2013-33522-21091 to BY and S-zF) and the Crop Bioengineering Center of Iowa State University (S-zF).

## Conflict of Interest

The authors declare that the research was conducted in the absence of any commercial or financial relationships that could be construed as a potential conflict of interest.

## Supplementary Material

Supplementary Figure 1 Micropropagation of switchgrass. Longitudinally split nodal segments were cultured on the MS-0 medium without plant growth regulators.

Supplementary Figure 2 Outgrowth of the lowest axillary bud in mutant plants and the wild-type plants. Arrow indicates a tiller developing from the lowest node in the *Pvtb1a-Pvtb1b* mutant (52-1-3, aabb), which is usually absent in the WT. The WT plant was about 2 weeks older than the mutant plant. Other tillers were removed for better view.

Supplementary Figure 3 Estimation of allelic composition of *Pvtb1* genes in cDNA samples of the mutant 52-1.

Supplementary Figure 4 Mean-Difference plot showing the log-fold change and average abundance of each gene. Significantly up- and down-regulated genes in the mutant are highlighted in red and blue, respectively.

Supplementary Figure 5 Expression patterns of six genes in both qRT-PCR and RNA-seq results. Three independent biological replicates and technique replicates were applied for each qRT-PCR assay.
