# Supplementary figures and images for: Functional Analysis of the teosinte branched 1 Gene in the Tetraploid Switchgrass (Panicum virgatum L.) by CRISPR/Cas9-Directed Mutagenesis
Source: Front Plant Sci. 2020 Sep 23;11:572193. doi: 10.3389/fpls.2020.572193 (PMC7546813; doi:10.3389/fpls.2020.572193)

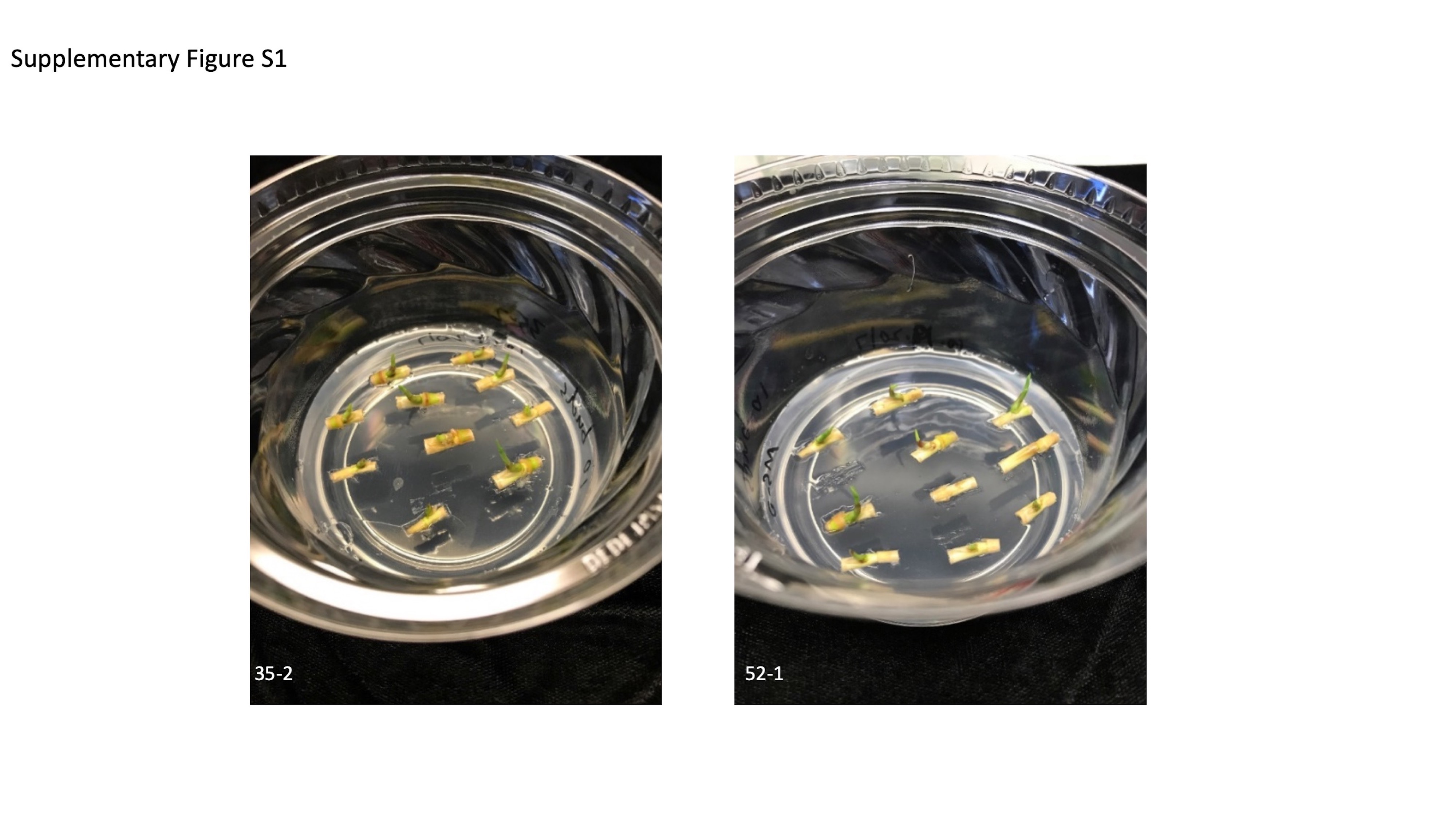

Supplement: Supplementary Figure 1 — Micropropagation of switchgrass. Longitudinally split nodal segments were cultured on the MS-0 medium without plant growth regulators. [file Image_1.jpeg]

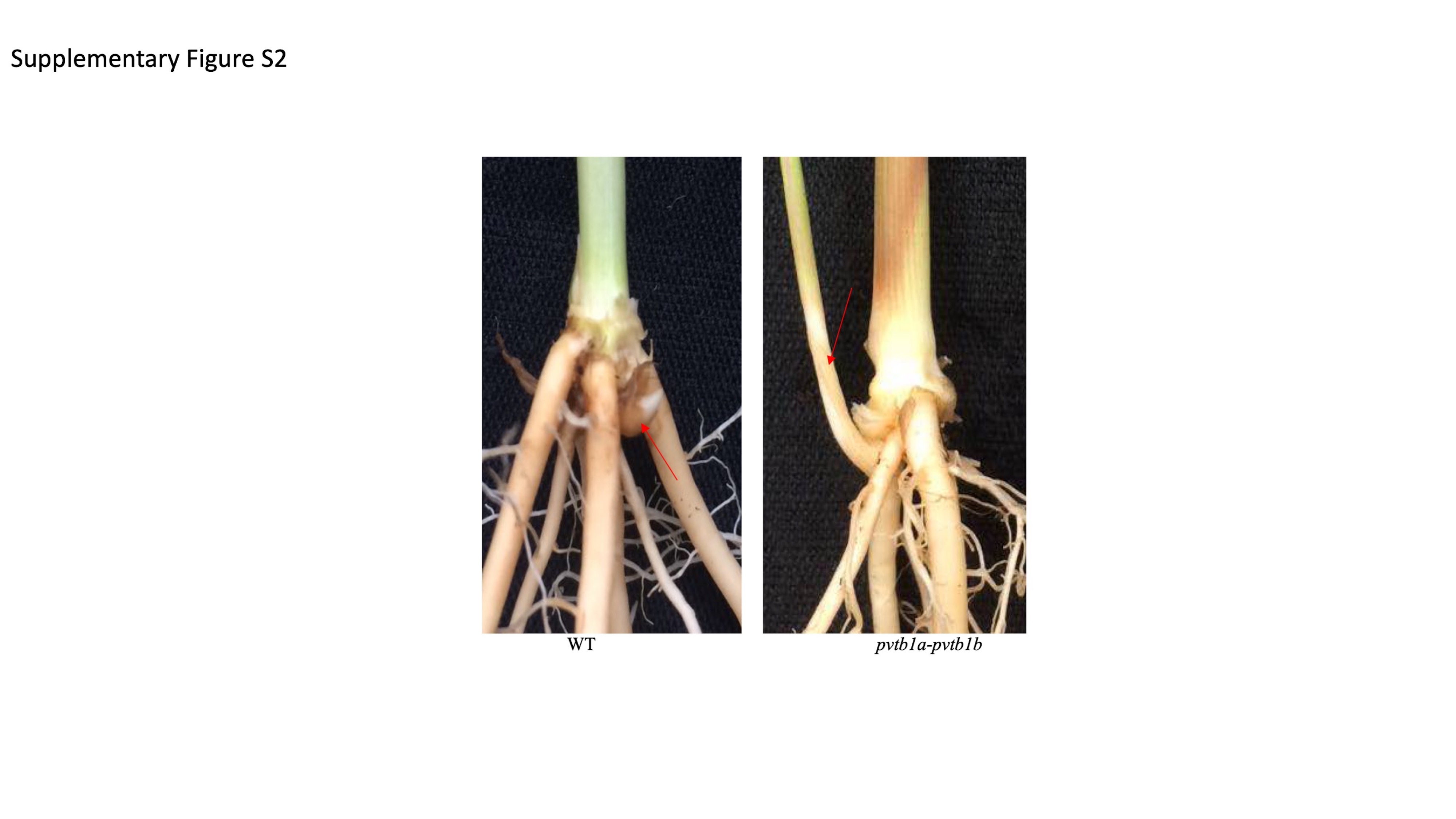

Supplement: Supplementary Figure 2 — Outgrowth of the lowest axillary bud in mutant plants and the wild-type plants. Arrow indicates a tiller developing from the lowest node in the Pvtb1a-Pvtb1b mutant (52-1-3, aabb), which is usually absent in the WT. The WT plant was about 2 weeks older than the mutant plant. Other tillers were removed for better view. [file Image_2.jpeg]

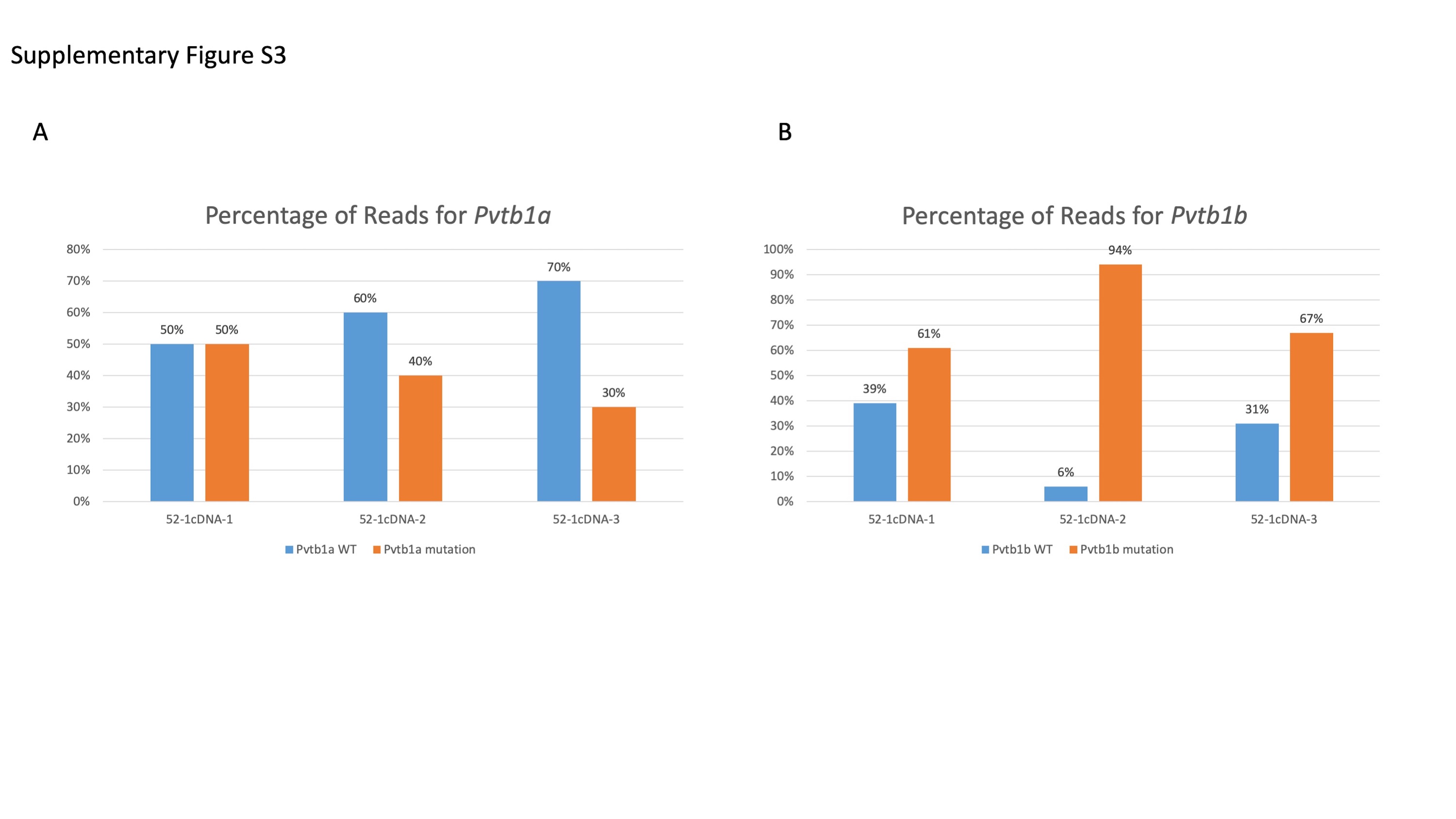

Supplement: Supplementary Figure 3 — Estimation of allelic composition of Pvtb1 genes in cDNA samples of the mutant 52-1. [file Image_3.jpeg]

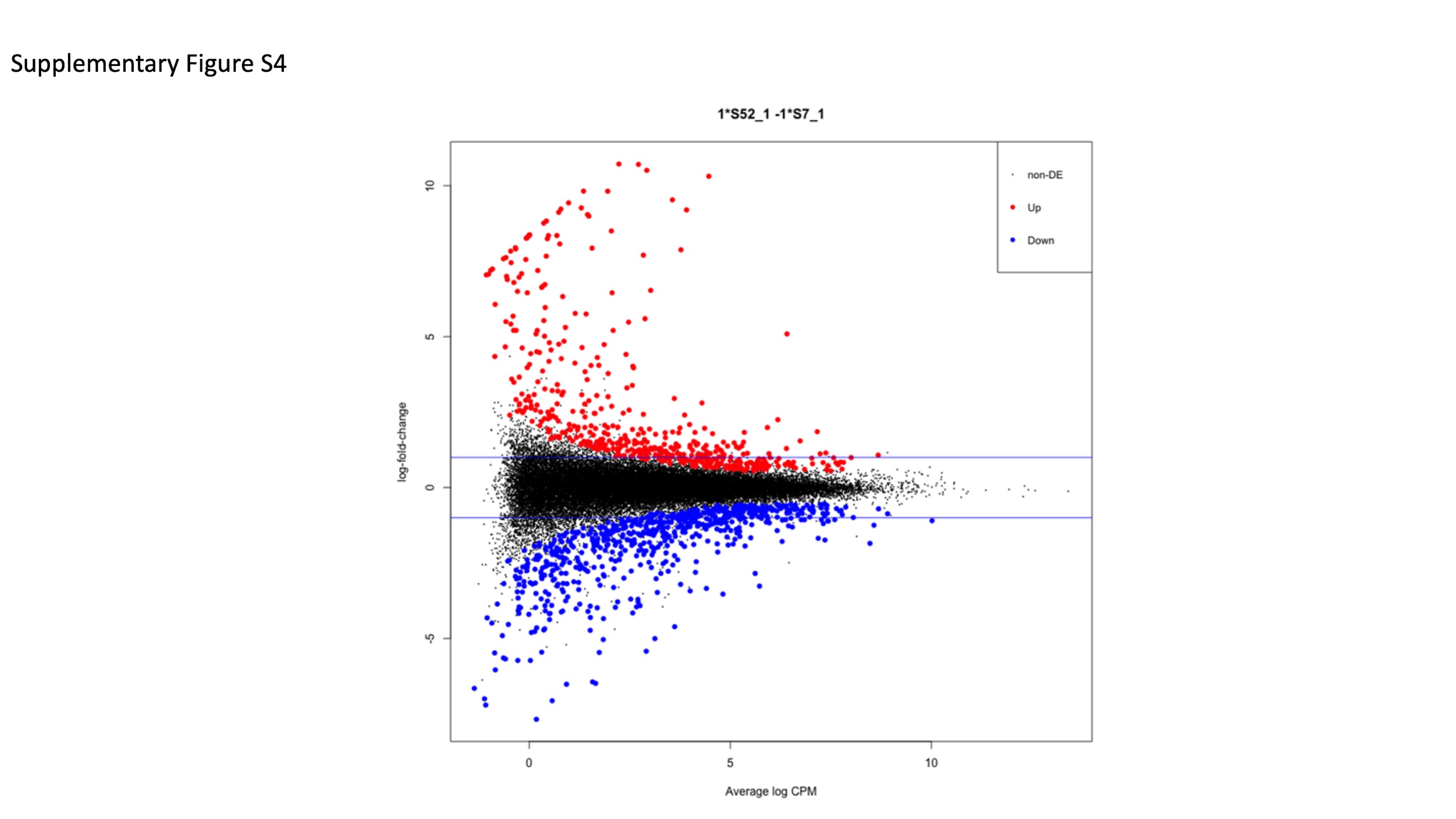

Supplement: Supplementary Figure 4 — Mean-Difference plot showing the log-fold change and average abundance of each gene. Significantly up- and down-regulated genes in the mutant are highlighted in red and blue, respectively. [file Image_4.jpeg]

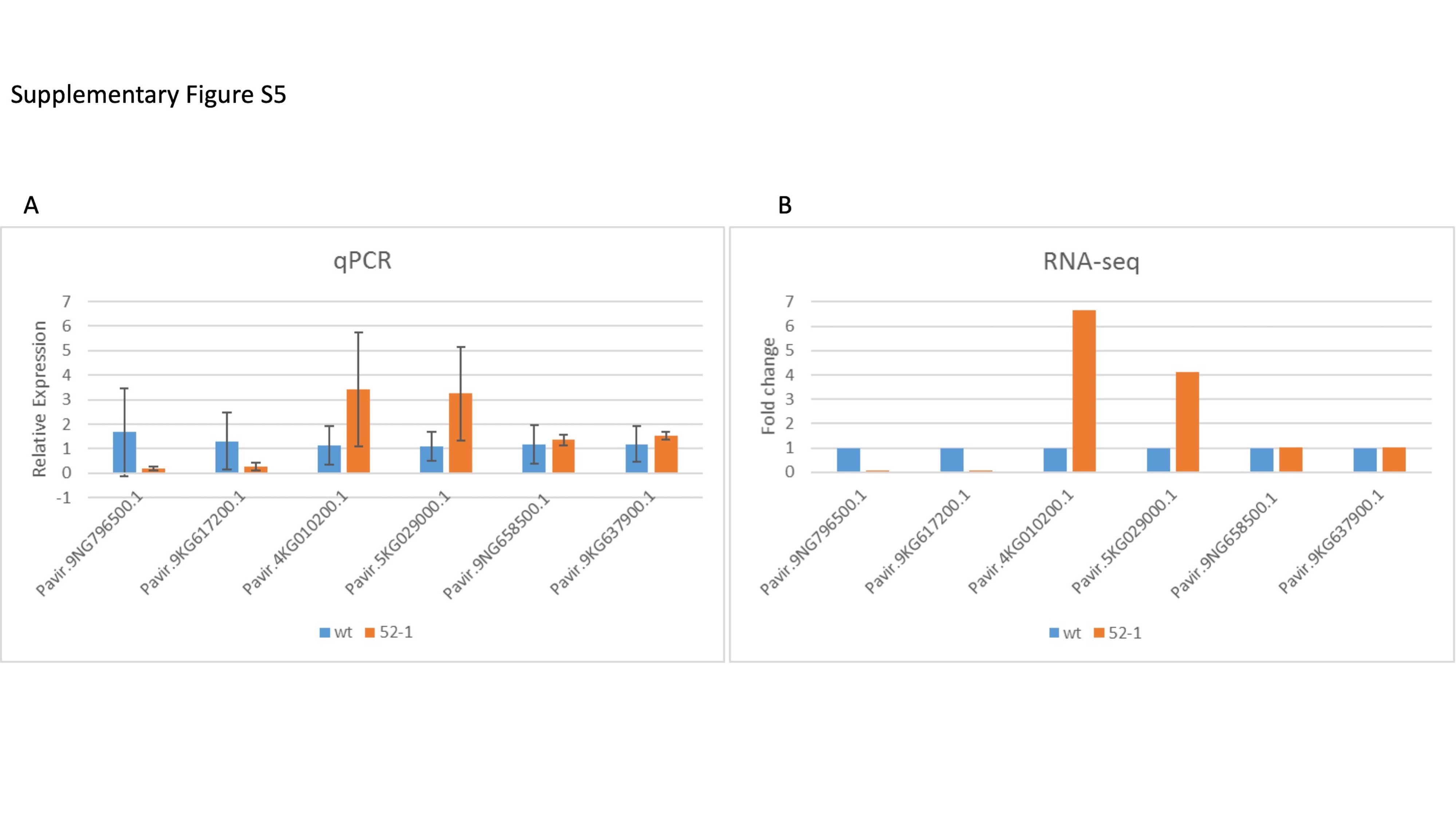

Supplement: Supplementary Figure 5 — Expression patterns of six genes in both qRT-PCR and RNA-seq results. Three independent biological replicates and technique replicates were applied for each qRT-PCR assay. [file Image_5.jpeg]
