# Supplementary material for: Functional Analysis of the teosinte branched 1 Gene in the Tetraploid Switchgrass (Panicum virgatum L.) by CRISPR/Cas9-Directed Mutagenesis
Source: Front Plant Sci. 2020 Sep 23;11:572193. doi: 10.3389/fpls.2020.572193 (PMC7546813; doi:10.3389/fpls.2020.572193)
Supplement: Supplementary file 6 [file Table_1.docx]

Supplementary_Table_S1. Sequencing of the targeted regions of *Pvtb1* genes in BC1 transgene-free mutants.

| BC1 plants name (genotype) | Sequence of the target regions of *tb1* genes |
| --- | --- |
| 52-1-BC1-1 (AaBb) | tb1a **CCC**CATGGACTTACCGCTTTACC........................**CCA**CCTCAGCTACCAGCTCGGTA WT/WT  tb1a **CCC**CAT-GACTTACCGCTTTACC.........................**CCA**CCT***T***CAGCTACCAGCTCGGTA -1/+1  tb1b **CCC**CATGGACTTACCGCTTTACC........................**CCA**CCTCAGCTACCTGCTCGGTA WT/WT  tb1b **CTC**CATG--CTCA--GCTTTACC............................**CCA**CCTC***A***AGCTACCTGCTCGGTA Mix/+1 |
| 52-1-BC1-3 (AaBb) | tb1a **CCC**CATGGACTTACCGCTTTACC........................**CCA**CCTCAGCTACCAGCTCGGTA WT/WT  tb1a **CCC**CAT-GACTTACCGCTTTACC.........................**CCA**CCTCAGCTACCAGCTCGGTA -1/WT  tb1b **CCC**CATGGACTTACCGCTTTACC........................**CCA**CCTCAGCTACCTGCTCGGTA WT/WT  tb1b **CTC**CATG--CTCA--GCTTTACC............................**CCA**CCTC***A***AGCTACCTGCTCGGTA Mix/+1 |
| 52-1-BC1-5 (AaBb) | tb1a **CCC**CATGGACTTACCGCTTTACC........................**CCA**CCTCAGCTACCAGCTCGGTA WT/WT  tb1a **CCC**CAT-GACTTACCGCTTTACC.........................**CCA**CCT***T***CAGCTACCAGCTCGGTA -1/+1  tb1b **CCC**CATGGACTTACCGCTTTACC........................**CCA**CCTCAGCTACCTGCTCGGTA WT/WT  tb1b **CTC**CATG--CTCA--GCTTTACC............................**CCA**CCTC***A***AGCTACCTGCTCGGTA Mix/+1 |
| 52-1-BC1-17 (AaBb) | tb1a **CCC**CATGGACTTACCGCTTTACC........................**CCA**CCTCAGCTACCAGCTCGGTA WT/WT  tb1a **CCC**CAT-GACTTACCGCTTTACC.........................**CCA**CCTCAGCTACCAGCTCGGTA -1/WT  tb1b **CCC**CATGGACTTACCGCTTTACC........................**CCA**CCTCAGCTACCTGCTCGGTA WT/WT  tb1b **CCC**CAT-GACTTACCGCTTTACC........................**CCA**CCTCAGCTACCTGCTCGGTA -1/WT |
| 52-1-BC1-18 (AaBb) | tb1a **CCC**CATGGACTTACCGCTTTACC........................**CCA**CCTCAGCTACCAGCTCGGTA WT/WT  tb1a **CCC**CAT-GACTTACCGCTTTACC.........................**CCA**CCTCAGCTACCAGCTCGGTA -1/WT  tb1b **CCC**CATGGACTTACCGCTTTACC........................**CCA**CCTCAGCTACCTGCTCGGTA WT/WT  tb1b **CCC**CAT-GACTTACCGCTTTACC........................**CCA**CCTCAGCTACCTGCTCGGTA -1/WT |
| 52-1-BC1-24 (AaBb) | tb1a **CCC**CATGGACTTACCGCTTTACC........................**CCA**CCTCAGCTACCAGCTCGGTA WT/WT  tb1a **CCC**CATG-----------------------------------------------------AGCTACCAGCTCGGTA -128bp  tb1b **CCC**CATGGACTTACCGCTTTACC........................**CCA**CCTCAGCTACCTGCTCGGTA WT/WT  tb1b **CCC**CAT-GACTTACCGCTTTACC........................**CCA**CCTCAGCTACCTGCTCGGTA -1/WT |
| 52-1-BC1-28 (AaBb) | tb1a **CCC**CATGGACTTACCGCTTTACC........................**CCA**CCTCAGCTACCAGCTCGGTA WT/WT  tb1a **CCC**CAT-GACTTACCGCTTTACC.........................**CCA**CCTCAGCTACCAGCTCGGTA -1/WT  tb1b **CCC**CATGGACTTACCGCTTTACC........................**CCA**CCTCAGCTACCTGCTCGGTA WT/WT  tb1b **CTC**CATG--CTCA--GCTTTACC............................**CCA**CCTC***A***AGCTACCTGCTCGGTA Mix/+1 |
| 35-1-BC1-1 (AaBb) | tb1a **CCC**CATGGACTTACCGCTTTACC........................**CCA**CCTCAGCTACCAGCTCGGTA WT/WT  tb1a **CCC**CATG-----------------------------------------------------AGCTACCAGCTCGGTA -128bp  tb1b **CCC**CATGGACTTACCGCTTTACC........................**CCA**CCTCAGCTACCTGCTCGGTA WT/WT  tb1b **CCC**CATG-------------------------------------------------TCAGCTACCTGCTCGGTA -127bp |
| 52-1-BC1-7 (AABb) | tb1a **CCC**CATGGACTTACCGCTTTACC........................**CCA**CCTCAGCTACCAGCTCGGTA WT/WT  tb1b **CCC**CATGGACTTACCGCTTTACC........................**CCA**CCTCAGCTACCTGCTCGGTA WT/WT  tb1b **CTC**CATG--CTCA--GCTTTACC............................**CCA**CCTC***A***AGCTACCTGCTCGGTA Mix/+1 |
| 52-1-BC1-15 (AABb) | tb1a **CCC**CATGGACTTACCGCTTTACC........................**CCA**CCTCAGCTACCAGCTCGGTA WT/WT  tb1b **CCC**CATGGACTTACCGCTTTACC........................**CCA**CCTCAGCTACCTGCTCGGTA WT/WT  tb1b **CTC**CATG--CTCA--GCTTTACC............................**CCA**CCTC***A***AGCTACCTGCTCGGTA Mix/+1 |
| 52-1-BC1-19 (AABb) | tb1a **CCC**CATGGACTTACCGCTTTACC........................**CCA**CCTCAGCTACCAGCTCGGTA WT/WT  tb1b **CCC**CATGGACTTACCGCTTTACC........................**CCA**CCTCAGCTACCTGCTCGGTA WT/WT  tb1b **CCC**CAT-GACTTACCGCTTTACC........................**CCA**CCTCAGCTACCTGCTCGGTA -1/WT |
| 52-1-BC1-20 (AABb) | tb1a **CCC**CATGGACTTACCGCTTTACC........................**CCA**CCTCAGCTACCAGCTCGGTA WT/WT  tb1b **CCC**CATGGACTTACCGCTTTACC........................**CCA**CCTCAGCTACCTGCTCGGTA WT/WT  tb1b **CTC**CATG--CTCA--GCTTTACC............................**CCA**CCTC***A***AGCTACCTGCTCGGTA Mix/+1 |
| 52-1-BC1-22 (AABb) | tb1a **CCC**CATGGACTTACCGCTTTACC........................**CCA**CCTCAGCTACCAGCTCGGTA WT/WT  tb1b **CCC**CATGGACTTACCGCTTTACC........................**CCA**CCTCAGCTACCTGCTCGGTA WT/WT  tb1b **CTC**CATG--CTCA--GCTTTACC............................**CCA**CCTC***A***AGCTACCTGCTCGGTA Mix/+1 |
| 35-2-BC1-1 (AaBB) | tb1a **CCC**CATGGACTTACCGCTTTACC........................**CCA**CCTCAGCTACCAGCTCGGTA WT/WT  tb1a **CCC**CATGGACTTACCGCTTTACC........................**CCA**CCT-AGCTACCAGCTCGGTA WT/-1  tb1b **CCC**CATGGACTTACCGCTTTACC........................**CCA**CCTCAGCTACCTGCTCGGTA WT/WT |
| 35-2-BC1-6 (AaBB) | tb1a **CCC**CATGGACTTACCGCTTTACC........................**CCA**CCTCAGCTACCAGCTCGGTA WT/WT  tb1a **CCC**CATG-----------------------------------------------------AGCTACCAGCTCGGTA -128bp  tb1b **CCC**CATGGACTTACCGCTTTACC........................**CCA**CCTCAGCTACCTGCTCGGTA WT/WT |

Representative sequences of tb1 mutations induced by CRISPR/Cas9 with deletions (dashed lines), insertions (italic, bold letters) and substitutions (red letters). Sequences complementary to PAM sequence are in bold. Sequences between two target sites are indicated by black dots.
