# Supplementary material for: Functional Analysis of the teosinte branched 1 Gene in the Tetraploid Switchgrass (Panicum virgatum L.) by CRISPR/Cas9-Directed Mutagenesis
Source: Front Plant Sci. 2020 Sep 23;11:572193. doi: 10.3389/fpls.2020.572193 (PMC7546813; doi:10.3389/fpls.2020.572193)
Supplement: Supplementary file 11 [file Table_6.docx]

Table S6. Sequences of primers used in this study. The bold characters with underline indicates the Illumina overhang adapter sequences.

| **For NGS sequencing amplicons** | | |
| --- | --- | --- |
| Gene | Forward Primer (5’-3’) | Reverse Primer (5’-3’) |
| *tb1a* | **TCGTCGGCAGCGTCAGATGTGTATAAGAGACAG**GCCTTGGAGTCCCATCAGTAA | **GTCTCGTGGGCTCGGAGATGTGTATAAGAGACAG**GCGAGTCGATCACGGCTTGT |
| *tb1b* | **TCGTCGGCAGCGTCAGATGTGTATAAGAGACAG**CTTAGTGGCAGGACCTAGCG | **GTCTCGTGGGCTCGGAGATGTGTATAAGAGACAG**ACGGCGAGTCGATCACG |
| **For Transgene detection** | | |
|  | gRNA (Forward Primer) | OsCas9 promoter (Reverse Primer) |
| gRNA/Cas9 | GTGTGGTAAAGCGGTAAGTCCATG | CCTGTTGTCAAAATACTCAA |
| **For gene isolation and Sanger sequencing** | | |
| Gene | Forward Primer (5’-3’) | Reverse Primer(5’-3’) |
| *Pvtb1a* | TGCCGCTCTCTCACATTCAC | GTGCATATCTTGCTGTGCCG |
| *Pvtb1b* | CTTAGTGGCAGGACCTAGCG | AGTTCAACATCACGCGGTCT |
| M13 | TGTAAAACGACGGCCAGT | CAGGAAACAGCTATGACC |
| **For qRT-PCR** | | |
| Gene | Forward Primer (5’-3’) | Reverse Primer(5’-3’) |
| *PvUbiquitin* | TTCGTGGTGGCCAGTAAG | AGAGACCAGAAGACCCAGGTACAG |
| Pavir.9KG637900.1 | TCAGTGTCCTGACGAGAATTAAG | CAGAGATCACCAGTCCATGATAAG |
| Pavir.9NG658500.1 | GTGCAGGAGATCAGAAAGGATTA | ACTACTGACTTGGTTGCTACTG |
| Pavir.9NG796500.1 | GTGATTTAATTGGGCGGGTTC | CTCTACAATTCTCCGTCTCTGTAAG |
| Pavir.9KG617200.1 | GCATTTGCTCATTGCTCTTACA | TGTTCGATCTCCCATCTTTCTATC |
| Pavir.5KG029000.1 | CAACCTTTACATCTGGGAGGAG | TTGACTCATCAGGCTAGTCATTT |
| Pavir.4KG010200.1 | TGCTGAACGAGAAGGTGAAATA | CTACCGTACCGCATGAAAGAA |
